# Antitumor properties of Coenzyme Q_0_ against human ovarian carcinoma cells *via* induction of ROS-mediated apoptosis and cytoprotective autophagy

**DOI:** 10.1038/s41598-017-08659-7

**Published:** 2017-08-14

**Authors:** You-Cheng Hseu, Tai-Jung Tsai, Mallikarjuna Korivi, Jer-Yuh Liu, Hui-Jye Chen, Chung-Ming Lin, Yi-Chun Shen, Hsin-Ling Yang

**Affiliations:** 1 Department of Cosmeceutics, College of Biopharmaceutical and Food Sciences, China Medical University, Taichung, 40402 Taiwan; 2 Department of Health and Nutrition Biotechnology, Asia University, Taichung, 41354 Taiwan; 3 Institute of Nutrition, College of Biopharmaceutical and Food Sciences, China Medical University, Taichung, 40402 Taiwan; 4 Graduate Institute of Cancer Biology, China Medical University, Taichung, 40402 Taiwan; 5 Graduate Institute of Basic Medical Science, China Medical University, Taichung, 402 Taiwan; 6 Department of Biotechnology, Ming Chuan University, Taoyuan, 333 Taiwan

## Abstract

Coenzyme Q_0_ (CoQ_0_, 2,3-dimethoxy-5-methyl-1,4-benzoquinone) has been reported to exert anticancer properties against human breast/lung cancer cells. This study investigated the *in vitro* and *in vivo* anticancer properties of CoQ_0_ on human ovarian carcinoma (SKOV-3) cells and xenografted nude mice, and revealed the underlying molecular mechanism. CoQ_0_ induced G_2_/M arrest through downregulation of cyclin B1/A and CDK1/K2 expressions. CoQ_0_-induced autophagy as a survival mechanism was evidenced by increased accumulation of LC3-II, GFP-LC3 puncta, AVOs formation and Beclin-1/Bcl-2 dysregulation. Increased TUNEL-positive cells and Annexin-V/PI stained cells indicated CoQ_0_-induced late apoptosis. Both mitochondrial (caspase-3, PARP and Bax/Bcl-2 dysregulation) and ER stress (caspase-12 and Hsp70) signals are involved in execution of apoptosis. Interestingly, CoQ_0_-induced apoptosis/autophagy is associated with suppression of HER-2/*neu* and PI_3_K/AKT signalling cascades. CoQ_0_ triggered intracellular ROS production, whereas antioxidant *N*-acetylcysteine prevented CoQ_0_-induced apoptosis, but not autophagy. Inhibition of apoptosis by Z-VAD-FMK suppressed CoQ_0_-induced autophagy (diminished LC3-II/AVOs), indicates CoQ_0_-induced apoptosis led to evoke autophagy. Contrary, inhibition of autophagy by 3-MA/CQ potentiated CoQ_0_-induced apoptosis (increased DNA fragmentation/PARP cleavage). Furthermore, CoQ_0_ treatment to SKOV-3 xenografted nude mice reduced tumor incidence and burden. Histopathological analyses confirmed that CoQ_0_ modulated xenografted tumor progression by apoptosis induction. Our findings emphasize that CoQ_0_ triggered ROS-mediated apoptosis and cytoprotective autophagy.

## Introduction

Cancer is one of the pathological scenarios where apoptosis is inactivated, resulting in accumulation of malignant cells that will not die. Ovarian cancer is the seventh most common cancer in women worldwide, which accounts for nearly 4% of all new cases of cancer in women. This lethal malignancy usually diagnosed at later stage with a 5 years survival rate of below 30%. Since most of the ovarian cancer cases are diagnosed after wide spreading of tumors within the peritoneal cavity, it is restraining the effectiveness of chemotherapy, and associated death is believed to be therapy-resistant metastasis^1, 2^. The prognosis is usually poor for ovarian cancer patients, because the disease reaching an advanced stage before it is discovered^3^. Despite overall declining death rates for cervical and uterus cancers, the annual report of ovarian cancer mortality has risen by 250% since 1930^4^. Primary cytoreductive surgery followed by chemotherapy with anticancer agents is the standard treatment regimen for patients with ovarian cancer, which improved survivals. Nevertheless, most of the patients with advanced cancer will eventually relapse and die of their cancer^2, 5^. A preliminary study showed improved efficacy of chemotherapy and normalized cancer biomarkers following a high-dose of antioxidant (containing coenzyme Q_10_) treatment with chemotherapy in patients with advanced ovarian cancer^6^. Management of ovarian cancers is still in high demand for effective therapy without adverse effects caused by the therapeutic agents.

Despite being the cause of problem, apoptosis or programmed cell death plays an important role in treatment of cancers, as it is a popular target of many therapeutic strategies. Mounting evidence revealed that induction of apoptosis by chemical substances or pro-apoptotic agents eventually controlled the spreading of cancer^7–9^. Apoptotic signals are reported to be triggered by several cellular events, including excessive production of reactive oxygen species (ROS), mitochondrial dysfunction and ER stress^8, 10, 11^. The apoptotic signals either intrinsic or extrinsic cascades lead to activate the caspases, a class of cysteine proteases that cleave different substrates and ultimately leading to cell dismantling and DNA fragmentation^12, 13^. In apoptosis, caspases can also cleave Beclin-1, an autophagic protein, and inhibit its pro-autophagic activity. Autophagy, a process of programmed cell survival or an adaptive response is activated during periods of cellular distress and extinguished during the cell-cycle^14^. At the molecular level, autophagy is regulated by a battery of signals, such as microtubule-associated protein light chain-3 (LC3), mammalian target of rapamycin (mTOR), autophagy-related protein 7 (ATG7) and Beclin-1. In addition, formation of acidic vesicular organelles (AVOs) is a hallmark of autophagy^15, 16^. Some anticancer agents that can induce apoptosis also stimulate autophagy as a death mechanism^17^. Recent developments in cancer research suggest that autophagy could be an additional target for adjuvant anticancer treatment, and inhibition of autophagy could strengthen the therapeutic efficacy of cancer treatment^18, 19^. However, whether autophagy promotes or inhibits cancer cell death in response to cellular stress is controversial. Indeed, there is a crosstalk between autophagy and apoptosis as they share common stimuli and signalling pathways^14, 20, 21^. Thus, understanding of the complex relation between apoptosis and autophagy in cancer therapy could enhance the treatment effectiveness.

Coenzyme Q (CoQ) is a well-known biomolecules comprised of a quinone nucleus and a hydrophobic side chain containing variable number of *trans*-isoprenoid units. CoQ_10_ is the major naturally occurring form of CoQ that containing 10 isoprenoid units, while CoQ_0_ is the novel analog without isoprenoid side chains^22^. Various CoQ analogs or ubiquinone (Ub) have been reported to either increase or decrease the production of ROS, and involved in opening/closing of mitochondrial permeability transition pore (PTP), which relies on cellular context^8, 23^. CoQ analogs with shorter isoprenoid side chains (CoQ_2_ and CoQ_4_) reported to induce apoptosis in mutated BALL-1 cells, but not longer isoprenoid chains (CoQ_6_ and CoQ_10_)^24^. Among various CoQ analogs (Ub_5_ and Ub_10_), CoQ_0_, a redox active compound profoundly triggered the ROS production in Clone-9 cells, induced PTP opening in cancerous rat liver MH1C1 cells and promote cell death^23^. CoQ_0_ has been shown as a potent cytotoxic compound towards human breast cancer cells by induction of apoptosis and cell-cycle arrest^25^. Our recent findings showed anti-angiogenic property of CoQ_0_ in stimulated human endothelial cells^26^.

Previously, we have shown the potent anticancer properties of *Antrodia camphorata* against human ovarian cancers via induction of apoptosis and cell-cycle arrest^27^. Several studies showed free radical scavenging or antioxidant activity^28, 29^ and anticancer properties of *Antrodia camphorata*, a well-known medicinal mushroom in Taiwan^30–32^. However, those studies are limited to claim the responsible bioactive compounds, and yet no study to demonstrate the anticancer properties of CoQ_0_, a key ingredient in *Antrodia camphorata*. In this study, we used CoQ_0_ that is isolated from *Antrodia camphorata*, and investigated its anticancer potentials in human ovarian cancer (SKOV-3) cells and xenografted nude mice. To distinguish the underlying molecular mechanisms, the effect of CoQ_0_ on ROS production; and the role of ROS on cancer cell survival, apoptosis and autophagy were examined by determining the key molecules involved in regulation of apoptosis and autophagy in SKOV-3 cells.

## Results

### CoQ_0_ inhibits viability and growth of human ovarian carcinoma cells

**Figure 1:**
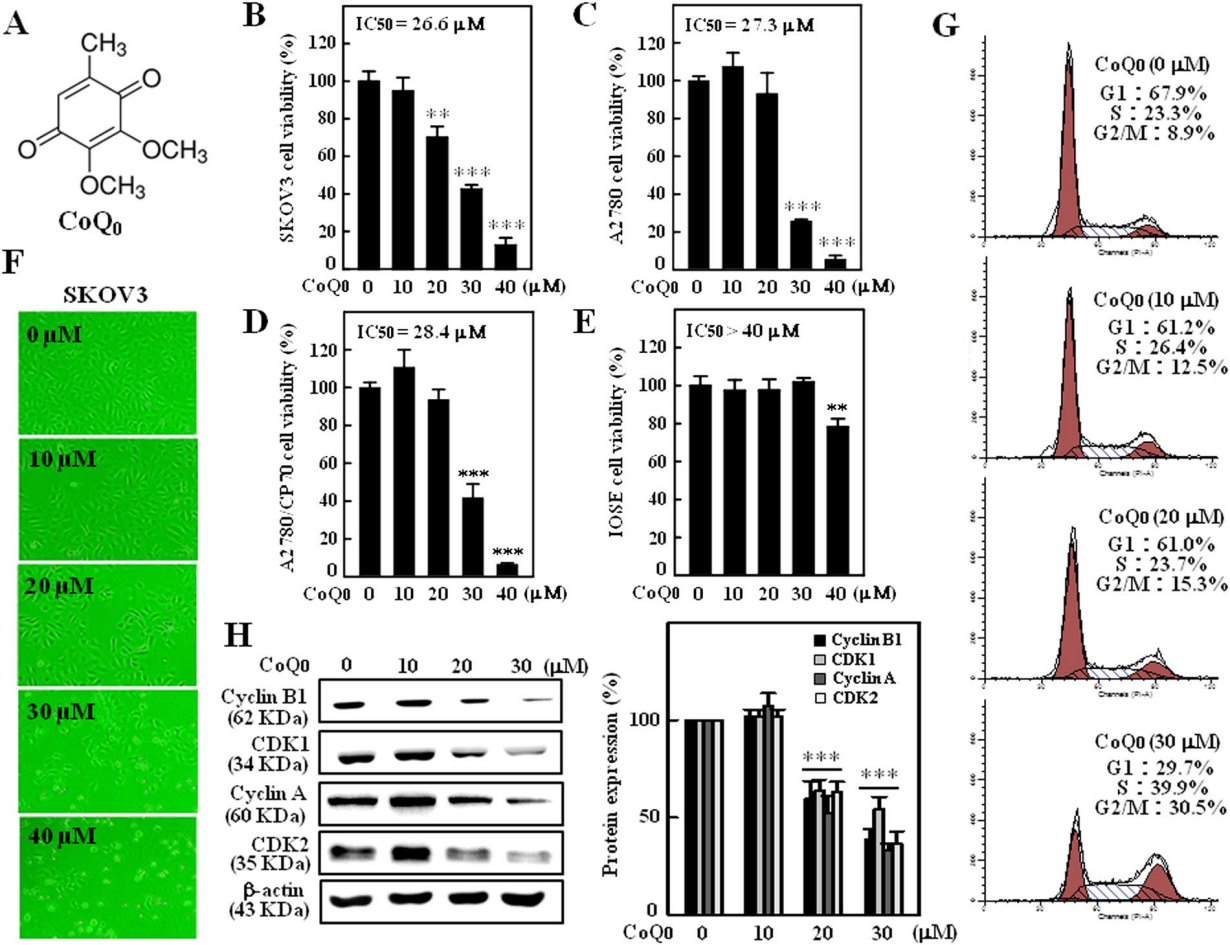
CoQ_0_ inhibits growth of human ovarian carcinoma cells and induces G2/M cell-cycle arrest in SKOV-3 cells. (**A**) Structure of CoQ_0_. (**B–D**) Human ovarian carcinoma **(**SKOV-3, A2870 and A2870/CP-70) and **(E)** human ovarian surface epithelial (IOSE) cells were treated with increasing concentrations of CoQ_0_ (0–40 µM) for 24 h. Cell viability was determined using MTT assay. (**F**) Morphological changes in CoQ_0_-treated (0–40 µM, 24 h) SKOV-3 cells were examined by phase-contrast microscope (200 × magnification). (**G**) SKOV-3 cells were treated with CoQ_0_ (0–30 µM) for 24 h, stained with PI and analyzed for cell-cycle phase using flow cytometry. The cellular distributions (percentage) in different phases of cell-cycle (G1, S and G2/M) were determined after CoQ_0_ treatment. Flow cytometry images shown here are from one representative analysis that was repeated three times with similar results. (**H**) SKOV-3 cells were treated with CoQ_0_ (0–30 µM) for 24 h, and cell-cycle regulatory proteins, cyclin B1, CDK1, cyclin A and CDK2 were examined using Western blot. Relative changes in protein intensities were quantified by commercially available software, and presented as histogram, control being as 1-fold. Results expressed as mean ± SD of three independent assays (n = 3), and significant at **p < 0.01; ***p < 0.001 compared with untreated control cells.

Prior to explore its anticancer properties, we examined the cytotoxic effects of CoQ_0_ on human ovarian carcinoma cell lines (SKOV-3, A2780 and A2870/CP70) and normal ovarian surface epithelial (IOSE) cells. Treatment of cells with increasing concentrations of CoQ_0_ (0–40 µM, 24 h) dose-dependently decreased viability of SKOV-3, A2780 and A2870/CP70 cells with the IC_50_ values of 26.6, 27.3 and 28.4 µM, respectively (Fig. 1B–D). The cytotoxic concentration of CoQ_0_ on normal IOSE cell lines is >40 µM (Fig. 1E). Inhibition of SKOV-3 cell viability with CoQ_0_ was prominent event at 20 µM concentration compared to A2780 and A2870/CP70 cell lines (Fig. 1B). Higher concentration of CoQ_0_ caused severe abrupt morphological changes, which were represented by cell shrinkage and decreased density of SKOV-3 cells (Fig. 1F). We assume that the cytotoxic effect of CoQ_0_ may be due to the induction of cell-cycle arrest.

### CoQ_0_ induces G_2_/M cell-cycle arrest and reduces cell-cycle proteins in SKOV-3 cells

To address whether CoQ_0_ induces cell-cycle arrest in SKOV-3 cells, we measured the distribution of cells (%) in different phases of cell-cycle following CoQ_0_ treatment (0–30 µM, 24 h). Flow cytometry data showed that CoQ_0_ resulted in a progressive and sustained accumulation of cells in G_2_/M phase, while cells in G_1_ phase were gradually decreased in a dose-dependent fashion. The accumulation of cells in G_2_/M phase is ~30.5% with 30 µM CoQ_0_, whereas cells in G_2_/M phase are only 8.9% without CoQ_0_ treatment (Fig. 1G). Concurrently, the expressions of cell-cycle regulatory proteins, including cyclin B1, cyclin dependent kinase 1 (CDK1), cyclin A and CDK2 were dose-dependently decreased with CoQ_0_ (Fig. 1H). These results explain that CoQ_0_ considerably inhibited ovarian cancer (SKOV-3) cell proliferation through induction of G_2_/M cell-cycle arrest and reduction of cell-cycle regulatory proteins.

### CoQ_0_ triggers intracellular ROS levels to promote SKOV-3 cell death

**Figure 2:**
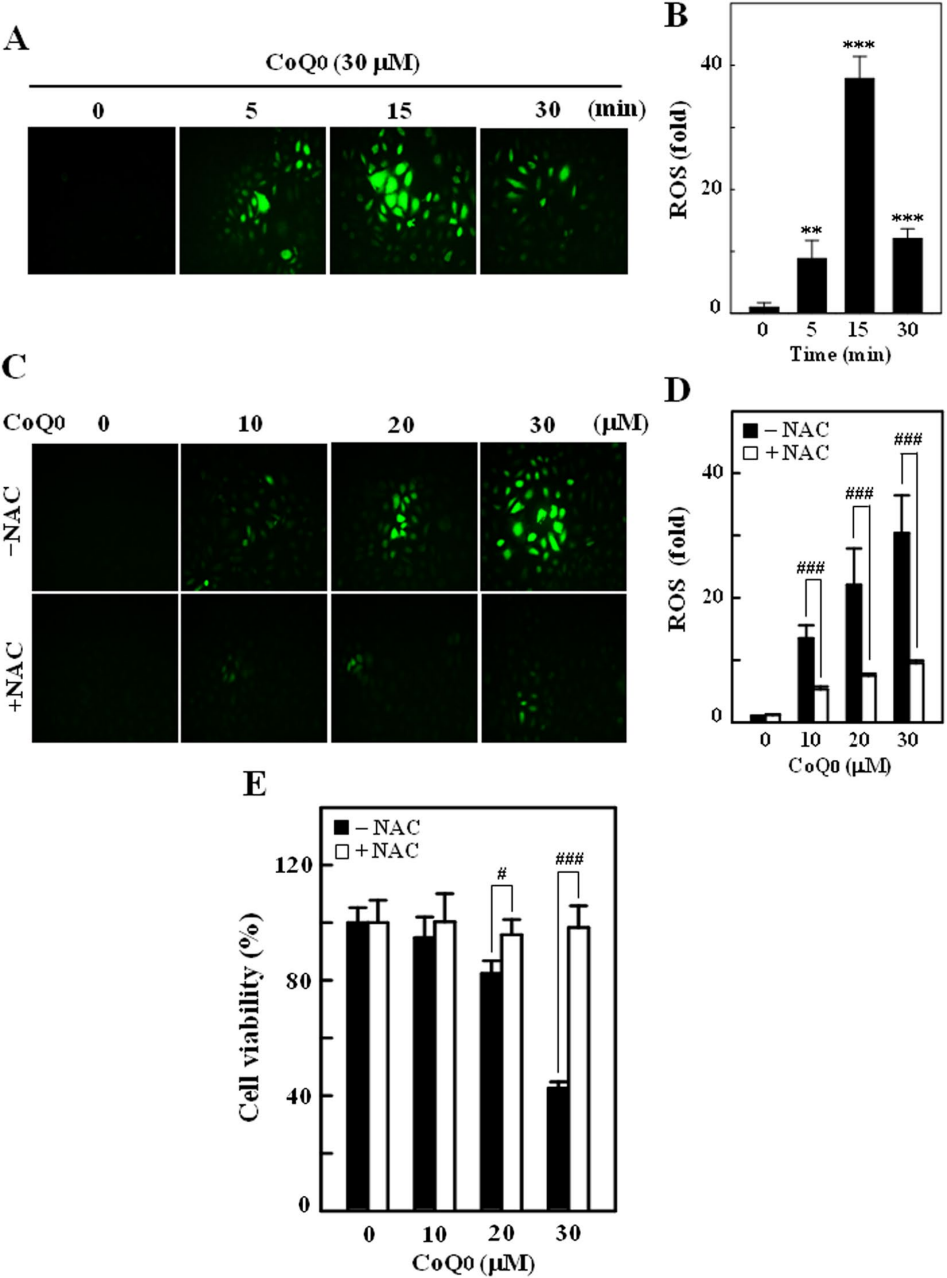
CoQ_0_ induces intracellular ROS generation in SKOV-3 cells. (**A**) Cells were treated with CoQ_0_ (30 µM) for 0–30 min and generation of intracellular ROS were measured using fluorescent microscopy (200 × magnification). The non fluorescent probe DCFH_2_-DA reacts with cellular ROS and metabolized into fluorescent DCF. (**B**) The fluorescence intensity of DCF-stained cells, represent the levels of ROS was quantified by Olympus Soft Imaging Solution, and presented as histogram. Results are significant at **p < 0.01; ***p < 0.001 compared to time 0 min. (**C**) Cells were treated with CoQ_0_ (10–30 µM) for 15 min, and ROS generation was measured in the presence or absence of ROS inhibitor (2 mM NAC, 1 h prior to CoQ_0_). The levels of intracellular ROS were indicated by strong or weak fluorescence intensity. (**D**) ROS levels were quantified and expressed in bar diagram as percentage of control. (**E**) Cells were treated with ROS inhibitor (NAC, 2 mM) 1 h prior to CoQ_0_ treatment (0–30 µM, 24 h), and cell viability of was determined by MTT assay. Results expressed as mean ± SD of three independent assays (n = 3), and significant at ^#^ *p* < 0.1; ^###^ *p* < 0.001 compared with CoQ_0_ alone treated cells.

Excessive generation of ROS by oxidants/chemical substances is reported to potentiate cancer cell death *via* apoptosis or autophagy mechanisms^33^. We found treatment of SKOV-3 cells with CoQ_0_ (30 µM) for 0–30 min remarkably increased the intracellular ROS levels. Especially, ROS levels at 15 min following CoQ_0_ treatment were significantly higher (~38 fold) than the control, as evidenced by increased dichlorofluorescein (DCF) (Fig. 2A and B). Interestingly, cells incubated with ROS inhibitor (N-acetylcysteine [NAC], 2 mM) 1 h prior to CoQ_0_ treatment (0–30 µM, 15 min) substantially inhibited the ROS production (Fig. 2C and D). We further demonstrated that NAC pretreatment completely reversed the CoQ_0_-induced death of SKOV-3 cells (Fig. 2E). These findings suggest that CoQ_0_ triggered intracellular ROS generation and that are possibly contribute to death of SKOV-3 cells.

### CoQ_0_ promotes LC3 accumulation and AVOs formation in SKOV-3 cells

**Figure 3:**
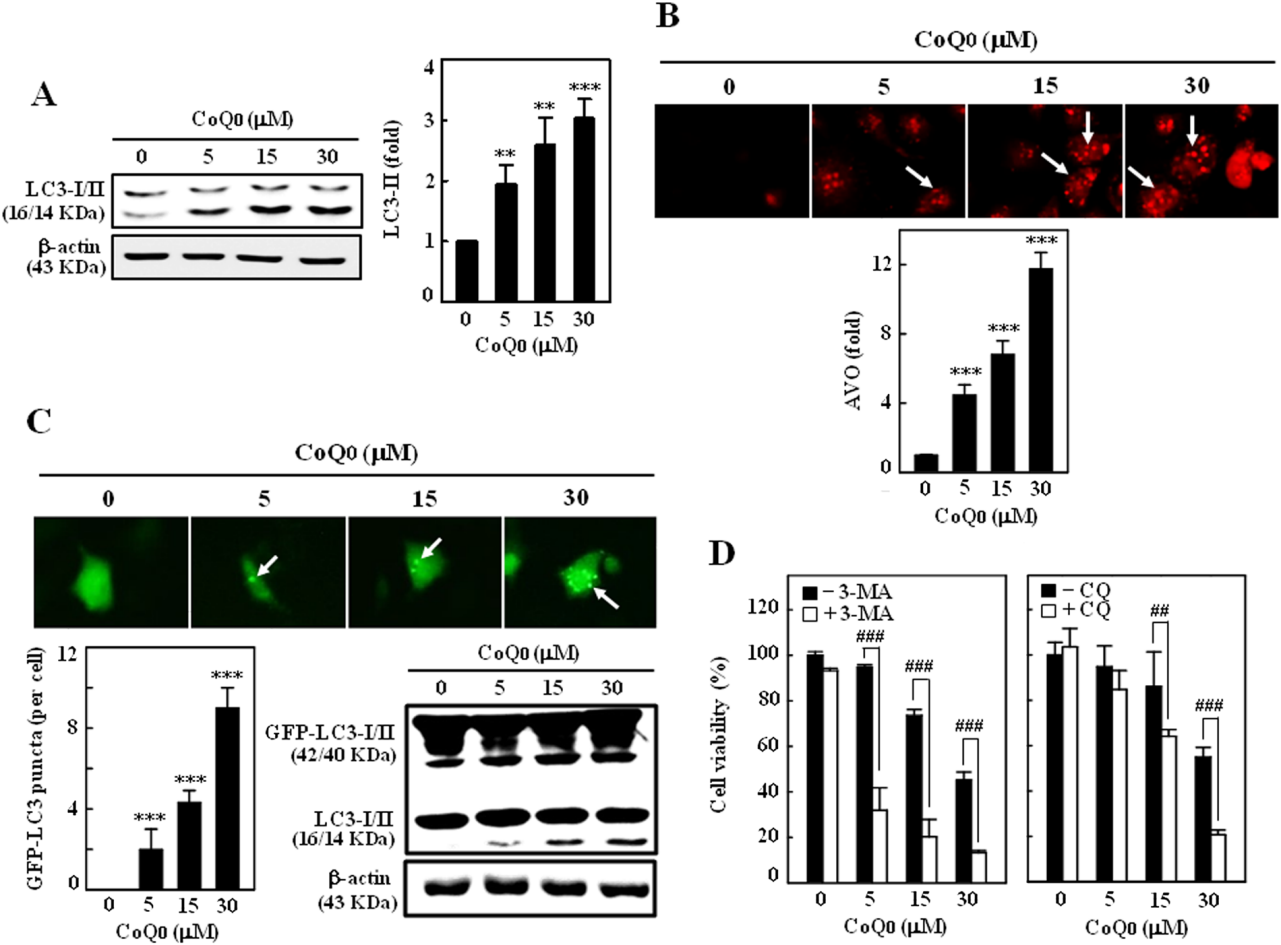
CoQ_0_ promotes cytoprotective autophagy as a survival mechanism in SKOV-3 cells. (**A**) Cells were treated with various concentrations of CoQ_0_ (0–30 µM) for 24 h and then conversion of LC3-I to LC3-II was determined by Western blot. Relative changes in the intensities of protein bands were quantified by commercially available quantitative software. (**B**) CoQ_0_ induces AVOs formation. Cells were treated with CoQ_0_ (0–30 µM) for 24 h and stained with AO. Formation of AVOs, represented by red fluorescence intensity (in lysosomes) was visualized under a red filter fluorescence microscope (100 × magnification). Number of AO stained cells was presented as histogram, control being as 1.0 fold. (**C**) CoQ_0_ promotes conversion of GFP-LC3. Cells were transfected with GFP-LC3 expression vector for 24 h, and then treated with CoQ_0_ (0–30 µM) for 24 h. GFP-LC3 dots in cells were observed under a confocal microscope (200 × magnification). Conversions of GFP-LC3 and endogenous LC3 were determined by Western blot. (**D**) Cells were treated with autophagy inhibitors (2 mM 3-MA or 10 μM CQ) for 1 h followed by CoQ_0_ (0–30 µM) for 24 h, and viability was assayed by MTT assay. Results expressed as mean ± SD of three independent assays (n = 3). Significant at **p < 0.01; ***p < 0.001 compared with untreated control, and significant at ^##^ *p* < 0.01; ^###^ *p* < 0.001 compared with CoQ_0_ alone treated cells.

LC3, a promising autophagy marker exists in LC3–1 (cytosolic) and LC3‐II (membrane bound) forms. The conversion of LC3–1 to LC3–1I or accumulation of LC3‐II is correlated with the extent of autophagosome formation or increased numbers of AVOs in cells^15, 16^. To address whether CoQ_0_ could induce autophagy, we measured the conversion of LC3–1 to LC3–1I and AVOs formation following CoQ_0_ treatment (0–30 µM). As presented in Fig. 3A, CoQ_0_ dose-dependently increased the LC3-II accumulation in SKOV-3 cells. The sequential effect of CoQ_0_ on AVOs formation was measured *via* fluorescence microscope using acridine orange (AO) stain. Arrows on images clearly indicating the increased appearance of AVOs (red fluorescence), following CoQ_0_ treatment (Fig. 3B). The high dose of CoQ_0_ (30 µM) resulted in large number of AVOs (>10 fold) (Fig. 3B) that is corresponding to the greater accumulation of LC3-II in SKOV-3 cells.

### CoQ_0_ enhances GFP-LC3 conversion in SKOV-3 cells

To confirm CoQ_0_-induced autophagy, GFP-LC3 plasmid was transiently transfected into the SKOV-3 cells, and conversion of GFP-LC3 and endogenous LC3 levels were monitored following CoQ_0_ treatment (0–30 µM, 24 h). Images from confocal microscopy depicts that CoQ_0_-treated cells represented by a cornucopia of green LC3 punctate dots in the cytoplasm, while the control cells showed a diffused and weak LC3 punctate dots (Fig. 3C). We demonstrated that CoQ_0_ increased both the percentage of cells with GFP-LC3 dots and the average numbers of GFP-LC3 dots per cell in a dose-dependent fashion (Fig. 3C). Western blot results further convinced that CoQ_0_ treatment significantly promoted the conversion of LC3–1 to LC3-II in SKOV-3 cells (Fig. 3C).

### CoQ_0_ activates autophagy as a survival mechanism in SKOV-3 cells

Autophagy has been claimed to play a paradoxical role in controlling of cell death and survival in response to various stimuli^34^. Since CoQ_0_ shown to activate autophagy in SKOV-3 cells, we wonder whether this autophagy could contribute to cell death or survival. To disclose this phenomenon, SKOV-3 cells were pretreated with autophagy inhibitors, 3-methyladenine (3-MA, inhibitor of early autophagy/LC3-II accumulation) or chloroquine (CQ, inhibitor of late autophagy/promoter of LC3-II accumulation), and cell viability was assayed following CoQ_0_ treatment (0–30 µM, 24 h). Interesting results showed that inhibition of autophagy by 3-MA (2 mM) or CQ (10 µM) didn’t suppress the CoQ_0_-mediated cell death, instead exacerbates the cell death (Fig. 3D). These findings imply that CoQ_0_-induced autophagy is not involved in death of SKOV-3 cells, which might be a cell survival mechanism.

### CoQ_0_ triggers apoptotic death of SKOV-3 cells via mitochondrial and ER-stress signals

**Figure 4:**
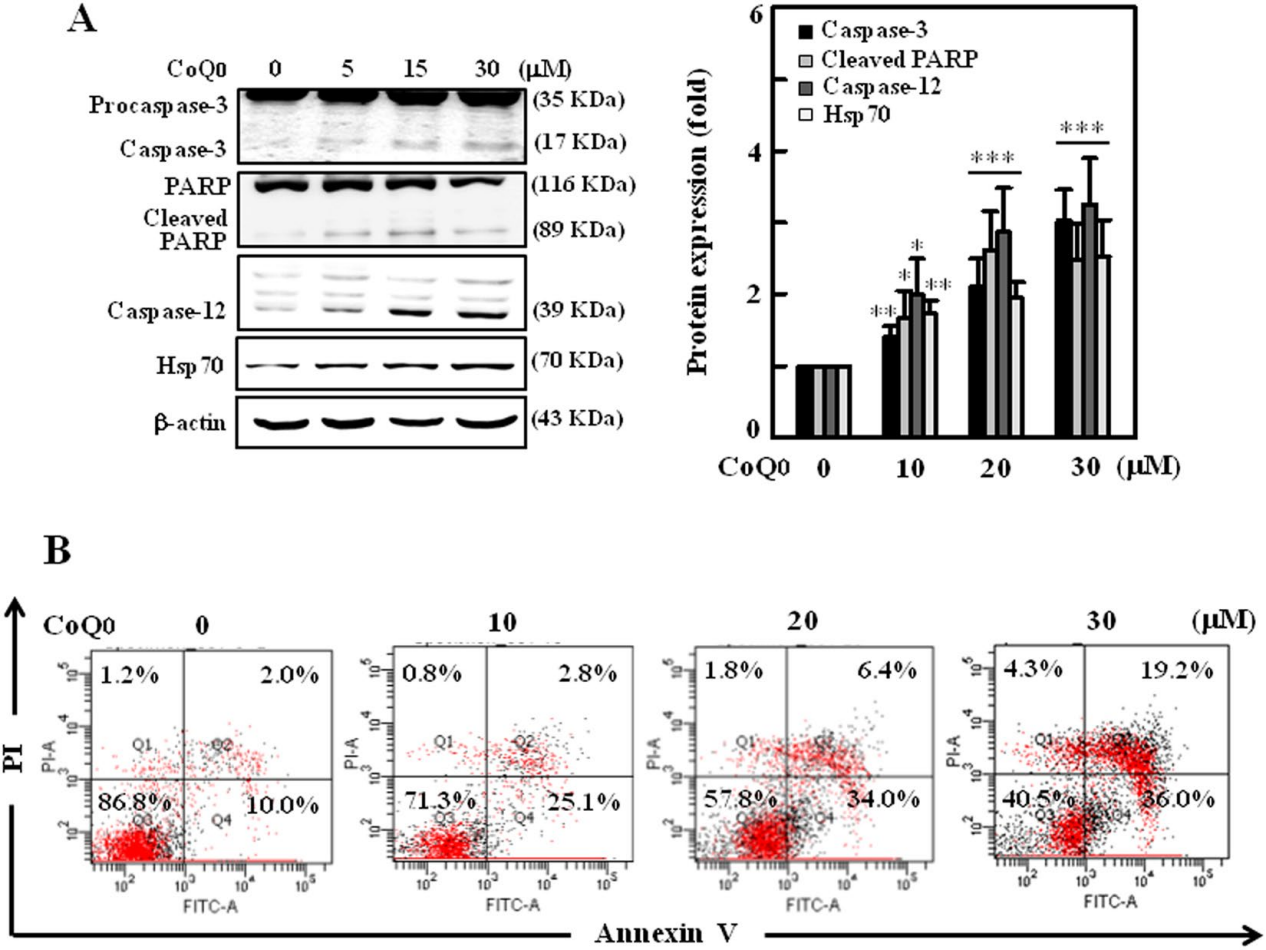
CoQ_0_ induces apoptosis through mitochondrial and ER stress pathways in SKOV-3 cells. (**A**) Cells were treated with CoQ_0_ (0–30 µM) for 24 h, and apoptotic proteins involved in mitochondrial pathway (procaspse-3, caspase-3 and PARP) and ER stress pathway (caspase-12 and Hsp70) were estimated by Western blot. Changes in protein intensities were quantified by commercially available software, and presented as histogram. **(B)** Annexin-V-FITC and PI staining was used to identify the early/late apoptosis or necrosis of cells followed by CoQ_0_ (0–30 µM) treatment. Results from flow cytometry analysis in each quadrant (Q) are labeled and interpreted as follows: (Q1) PI positive, Annexin-V-FITC-negative stained cells/necrosis. (Q2) PI positive, Annexin-V-FITC-positive stained cells/late apoptosis. (Q3) cells negative for both PI and Annexin-V-FITC staining/normal live cells. (Q4) PI-negative, Annexin-V-FITC-positive stained cells/early apoptosis. Results expressed as mean ± SD of three independent assays (n = 3), and significant at *p < 0.05; **p < 0.01; ***p < 0.001 compared with untreated control cells.

Apoptotic-cell death, a key strategic phenomenon in management of cancers is mediated by either mitochondrial or ER-stress signalling cascades^10, 11^. To explore the patterns of apoptosis induced by CoQ_0_ in SKOV-3 cells, the key molecular proteins involved in mitochondrial and ER-stress related apoptosis were determined by Western blot. As shown in Fig. 4A, procaspase-3, a highly expressed inactive form prior to CoQ_0_ incubation, was dose-dependently cleaved to active caspase-3 following CoQ_0_ treatment (0–30 µM, 24 h). It is known that caspase-3-mediated selective proteolytic cleavage of poly (ADP-ribose) polymerase (PARP) is a hallmark of apoptosis^35^. Thus, we measured the PARP levels in SKOV-3 cells, and found that CoQ_0_ increased the proteolytic cleavage of 116 kDa PARP to 89 kDa fragment (Fig. 4A). Next we investigated the effect of CoQ_0_ on ER-stress mediated apoptosis by detecting the changes of caspase-12 and heat shock protein-70 (HSP-70) that are implicated in ER-stress apoptosis^11^. We found CoQ_0_ (0–30 µM) remarkably increased caspase-12 and HSP-70 expressions in a dose-dependent manner (Fig. 4A). These findings suggest that CoQ_0_ activates both mitochondrial and ER-stress mediated apoptosis in SKOV-3 cells.

### CoQ_0_ promotes apoptosis in SKOV-3 cells

To address whether CoQ_0_ promotes apoptosis in SKOV-3 cells, next we performed Annexin-V- fluorescein isothiocyanate (FITC) and propidium iodide (PI) assay, which stain phosphatidylserine and DNA residues, respectively^36^. The flow cytometry results showed that treatment of SKOV-3 cells with CoQ_0_ (0–30 µM) dose-dependently increased the early and late apoptotic cells. The early apoptotic cells (Annexin-V-FITC-positive, PI-negative) represented in Q4 are 25.1%, 34% and 36% with 10, 20 and 30 µM CoQ_0_ treatments respectively (Fig. 4B).

### CoQ_0_ potentiates apoptotic DNA fragmentation and inhibition of apoptosis reversed death of SKOV-3 cells

**Figure 5:**
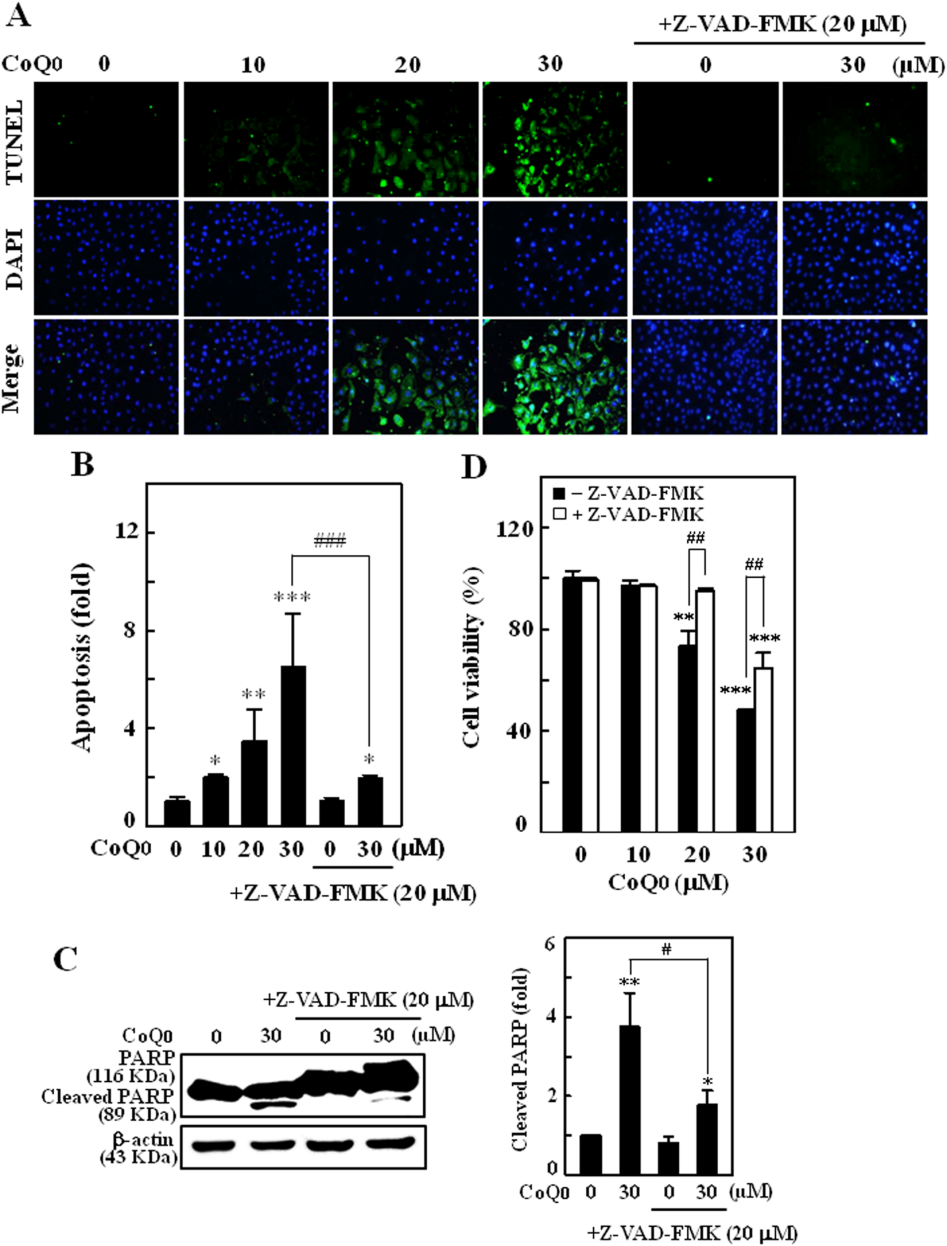
CoQ_0_ triggers apoptotic DNA fragmentation and promote death of SKOV-3 cells. (**A–D**) Cells were pretreated with caspase inhibitor (Z-VAD-FMK, 20 μM) for 1 h followed by CoQ_0_ (0–30 µM) for 24 h. **(A)** Apoptotic DNA fragmentation was determined by TUNEL assay. The green florescence indicates TUNEL-positive cells in the microscopic fields (200 × magnification) from three separate samples. **(B)** The fold of apoptotic cells was calculated by quantifying the florescence intensity using commercially available software. **(C)** Cleavage of PARP was estimated by Western blot. Changes in protein intensities were quantified by commercially available software. **(D)** Cell viability with or without Z-VAD-FMK treatment was determined by MTT assay. Values expressed as mean ± SD of three independent assays (n = 3). Significant at *p < 0.05; **p < 0.01; ***p < 0.001 compared with untreated control, and significant at ^##^ *p* < 0.01; ^###^ *p* < 0.001 compared with CoQ_0_ alone treated cells.

CoQ_0_-induced apoptotic DNA fragmentation was determined using TUNEL assay (terminal deoxynucleotidyl transferase dUTP nick end labeling). The fluorescent microscope images revealed that CoQ_0_ treatment (0–30 µM, 24 h) significantly increased the green fluorescence, TUNEL-positive cells (Fig. 5A), which denotes increased apoptotic DNA fragmentation. However, CoQ_0_-induced DNA fragmentation (~6-fold, 30 µM) was significantly (*P* < 0.001) diminished in cells pretreated with caspase inhibitor, Z-VAD-FMK (benzyloxycarbonyl-valyl-alanyl-aspartyl-[O-methyl]-fluoromethylketone, 20 μM) (Fig. 5A and B). Furthermore, CoQ_0_-induced PARP cleavage was also suppressed in the presence of Z-VAD-FMK, which indicates CoQ_0_ activates caspase mediated apoptosis in SKOV-3 cells (Fig. 5C). To confirm that CoQ_0_-induced death of SKOV-3 cell *via* activation of apoptosis, cells were pretreated with Z-VAD-FMK (20 μM, 1 h), and cell survival was assayed following CoQ_0_ treatment (0–30 µM, 24 h). We found that CoQ_0_-induced death of SKOV-3 cells was predominantly reversed by inhibition of apoptosis (Fig. 5D). These findings suggest that CoQ_0_ provoked apoptotic signals contribute to death of ovarian cancer cells.

### CoQ_0_ increases Beclin-1/Bcl-2 and Bax/Bcl-2, and inhibits HER-2/neu/AKT/mTOR signalling in SKOV-3 cells

**Figure 6:**
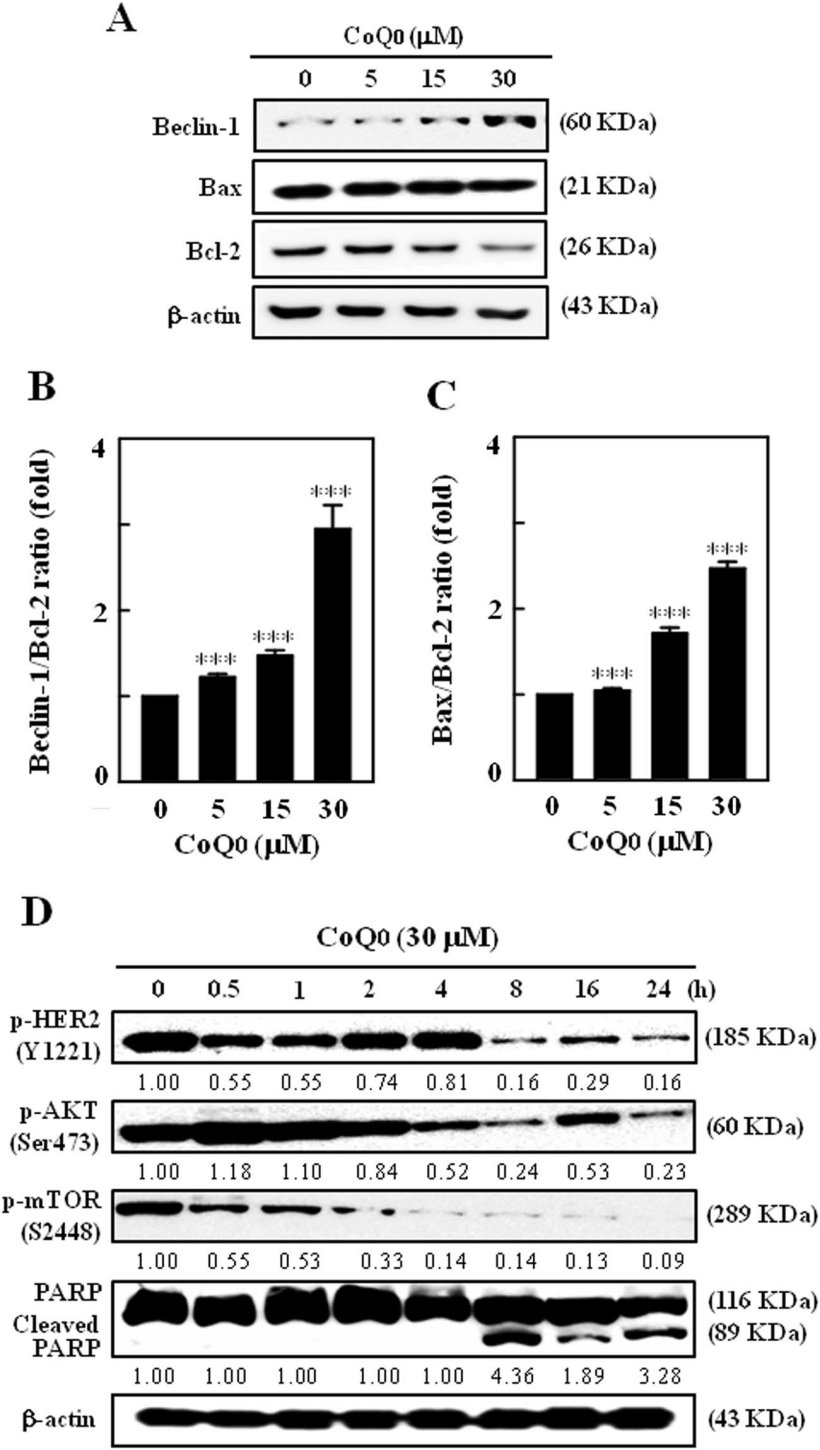
CoQ_0_ increases Beclin-1/Bcl-2 and Bax/Bcl-2 ratios, and inhibits HER-2/*neu*/AKT/mTOR signalling in SKOV-3 cells. **(A)** Dose-dependent effect of CoQ_0_ (0–30 µM, 24 h) on changes in Beclin-1, Bax and Bcl-2 proteins were determined by Western blot. Relative changes in the ratio of **(B)** Beclin-1/Bcl-2 and **(C)** Bax/Bcl-2 in accordance to dose were quantified by commercially available software, and presented as a histogram, control representing as 1.0 fold. Results expressed as mean ± SD of three independent assays (n = 3), and significant at ***p < 0.001 compared with untreated control cells. **(D)** Time-dependent effect of CoQ_0_ (0–24 h, 30 µM) on phosphorylation of HER-2/*neu* (Y1221), AKT (Ser473) and mTOR (S2448), and cleavage of PARP were determined by Western blot. Results expressed as mean ± SD of two independent assays (n = 2).

Since CoQ_0_ reported to induce apoptosis/autophagy in SKOV-3 cells, we studied the effect of CoQ_0_ (0–30 µM) on Bcl-2, and its role on Bax (pro-apoptotic) and Beclin-1 (pro-autophagic) expressions. Bcl-2 is reported to decrease the pro-autophagic property of Beclin-1, but Beclin-1 unable to neutralize the apoptotic function of Bcl-2^14^. In our study, we demonstrated that CoQ_0_ treatment substantially decreased the Bcl-2 expression, while increased the Beclin-1 and Bax expressions in a dose-dependent fashion (Fig. 6A). Degradation of Bcl-2 probably activates apoptosis in SKOV-3 cells. The dose-dependent increase of Bax/Bcl-2 ratio with CoQ_0_ represents the propagation of apoptotic mechanism in SKOV-3 cells (Fig. 6B and C).

Human epidermal growth factor receptor-2 (HER-2 *neu*) is a proto-oncogene implicated in malignant transformation, and overexpression of HER-2 has been found to aggressively promote the AKT/mTOR signals, which are responsible for regulation of tumor biology, including cancer cell invasion, differentiation and survival^37^. We found that CoQ_0_ treatment (30 µM, 0–24 h) downregulated the phosphorylated HER-2 (Y1221) levels. This decrease was accompanied with substantial time-dependent loss of p-AKT (Ser473) and p-mTOR (S2448) levels (Fig. 6D). CoQ_0_ enhanced the proteolytic cleavage of PARP (116 KDa to 89 KDa), which supports activated apoptotic signals in cancer cells (Fig. 6D). These results explain that CoQ_0_ may potentiate the apoptosis and/or autophagy mechanisms *via* suppression of HER-2/AKT/mTOR signalling cascades in SKOV-3 cells.

### Inhibition of ROS production obliterates CoQ_0_-induced apoptosis, but not autophagy in SKOV-3 cells

**Figure 7:**
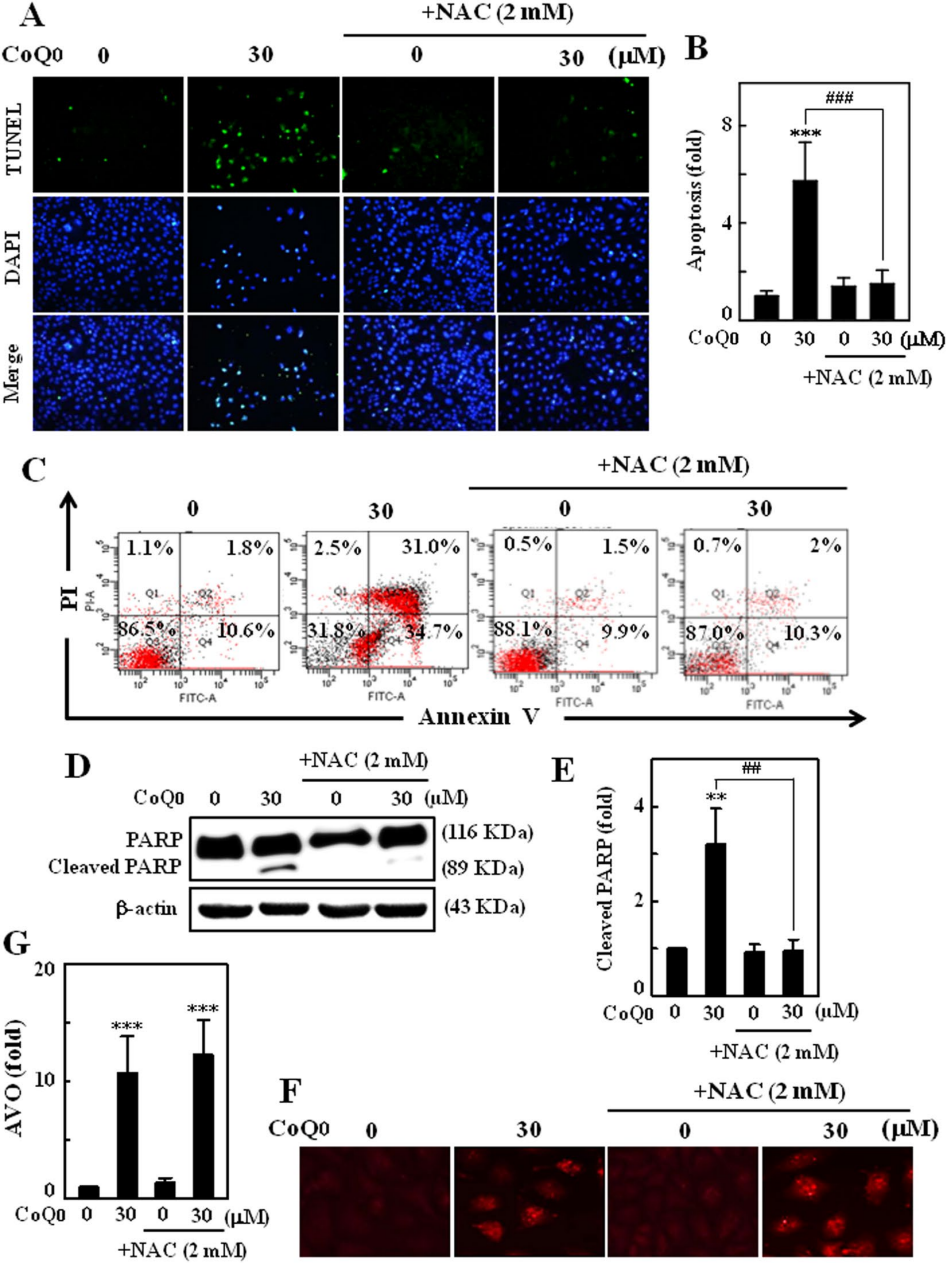
CoQ_0_-induced ROS involved in induction of SKOV-3 apoptosis, but not autophagy. **(A–G)** Cells were pretreated with NAC (2 mM) for 1 h, and then incubated with CoQ_0_ (30 µM) for 24 h. **(A)** Induction of apoptosis (DNA fragmentation) was determined by TUNEL assay in the presence or absence of NAC. The green florescence indicates TUNEL-positive cells in microscopic fields (200 × magnification). **(B)** The fold of apoptotic cells was calculated by measuring the florescence intensity using commercially available software. **(C)** Annexin-V-FITC and PI staining was used to identify the early/late apoptosis or necrosis of SKOV-3 cells. **(D)** Cleavage of PARP was monitored by Western blot. **(E)** Changes in protein intensities were quantified using commercially available software. **(F)** Formation of AVOs was visualized under a red filter fluorescence microscope (100 × magnification) using AO stain. **(G)** Number of AO stained cells with CoQ_0_ or NAC was presented as histogram, control being as 1.0 fold. Results expressed as mean ± SD of three independent assays (n = 3). Significant at **p < 0.01; ***p < 0.001 compared with untreated control, and significant at ^##^ *p* < 0.01; ^###^ *p* < 0.001 compared with CoQ_0_ alone treated cells.

Aberrant production of ROS involved in execution of apoptosis and/or autophagy^38, 39^. Since CoQ_0_ reported to trigger the ROS production and induce apoptosis in SKOV-3 cells, we hypothesized that CoQ_0_-induced ROS could propagate the apoptosis. To address this phenomenon, cells were pretreated with ROS inhibitor (NAC, 2 mM) for 1 h, and then incubated with CoQ_0_ (30 µM, 24 h). We found that tremendously increased apoptotic DNA fragmentation with CoQ_0_ was substantially diminished in the presence of NAC. The TUNEL positive cells with NAC are almost similar to that of control (Fig. 7A and B). CoQ_0_ provoked late (31%) and early (34.7%) apoptotic-cells visualized in Q2 and Q4 were remarkably attenuated by NAC pretreatment. This was indicated by reporting only 2% (Q2) and 10.3% (Q4) of late and early apoptotic-cells following blockade of ROS production (Fig. 7C). In addition, increased proteolytic cleavage of PARP by CoQ_0_ was obliterated in NAC pretreated cells (Fig. 7D and E). These results clearly demonstrating that CoQ_0_-induced ROS are involved in execution of SKOV-3 cell apoptosis.

Subsequently we examined the role of CoQ_0_-induced ROS on autophagy by detecting the AVOs in the presence or absence of NAC, following CoQ_0_ treatment. We found fascinating results that CoQ_0_-induced increased numbers of AVOs were not changed (remained same) even in the presence of NAC. This novel evidence explains that CoQ_0_-induced ROS are not involved in autophagy, or autophagy is not contributing to the death of cancer cells; despite apoptosis takes place (Fig. 7F and G).

### Inhibition of apoptosis suppresses CoQ_0_-induced autophagy in SKOV-3 cells

**Figure 8:**
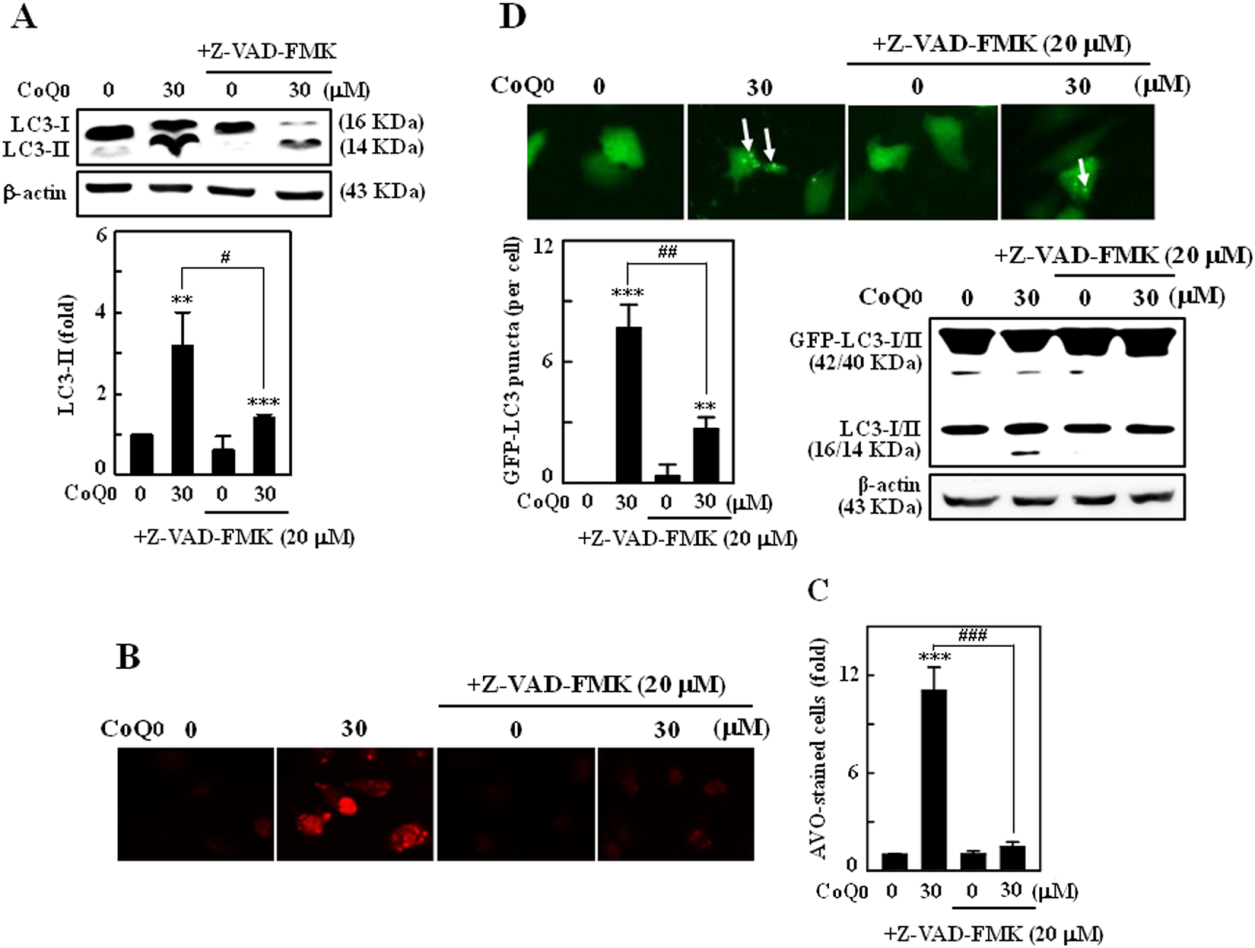
Inhibition of apoptosis suppressed CoQ_0_-induced autophagy in SKOV-3 cells. (**A–C**) Cells were pretreated with caspase inhibitor (Z-VAD-FMK, 20 μM) for 1 h, and then incubated with CoQ_0_ (30 µM) for 24 h. **(A)** LC3-I/II was determined using Western blot, and relative changes in protein intensities were quantified using commercially available software. **(B)** Formation of AVOs was visualized under a red filter fluorescence microscope (100 × magnification) using AO stain. **(C)** Number of AO stained cells with CoQ_0_ or Z-VAD-FMK treatment was presented as histogram, control being as 1.0 fold. **(D)** Cells were transfected with GFP-LC3 expression vector for 24 h before Z-VAD-FMK and CoQ_0_ treatment. GFP-LC3 dots in cells were observed under a confocal microscope (200 × magnification). Conversions of GFP-LC3 and endogenous LC3 were determined by Western blot. Results expressed as mean ± SD of three independent assays (n = 3). Significant at **p < 0.01; ***p < 0.001 compared with untreated control, and significant at ^#^ *p* < 0.05; ^##^ *p* < 0.01; ^###^ *p* < 0.001 compared with CoQ_0_ alone treated cells.

Next to address the role of apoptosis on autophagy under CoQ_0_ stimulation, SKOV-3 cells were treated with apoptosis inhibitor (Z-VAD-FMK, 20 μM) 1 h prior to CoQ_0_ incubation (30 µM, 24 h), and changes in LC3-II accumulation, conversion of GFP-LC3 and AVOs formation were monitored. We found that increased LC3-II accumulation in CoQ_0_ treated cells was noticeably limited by apoptosis inhibition (Fig. 8A). CoQ_0_-induced increased AVOs were substantially decreased in Z-VAD-FMK pretreated cells (Fig. 8B and C). Subsequently, Z-VAD-FMK pretreatment to the GFP-LC3 plasmid transfected cells, significantly diminished the CoQ_0_-induced conversion of GFP-LC3 and endogenous LC3 levels. This was evidenced by a dearth of green LC3 punctate dots and diffused fluorescence intensity in Z-VAD-FMK pretreated cells (Fig. 8D). CoQ_0_ promoted conversion of LC3–1 to LC3-II was also impaired by apoptosis inhibitor (Fig. 8D). Our experimental evidence reveals that CoQ_0_-induced apoptosis led to evoke the autophagy in SKOV-3 cells.

### Inhibition of autophagy promotes apoptosis in CoQ_0_-treated SKOV-3 cells

**Figure 9:**
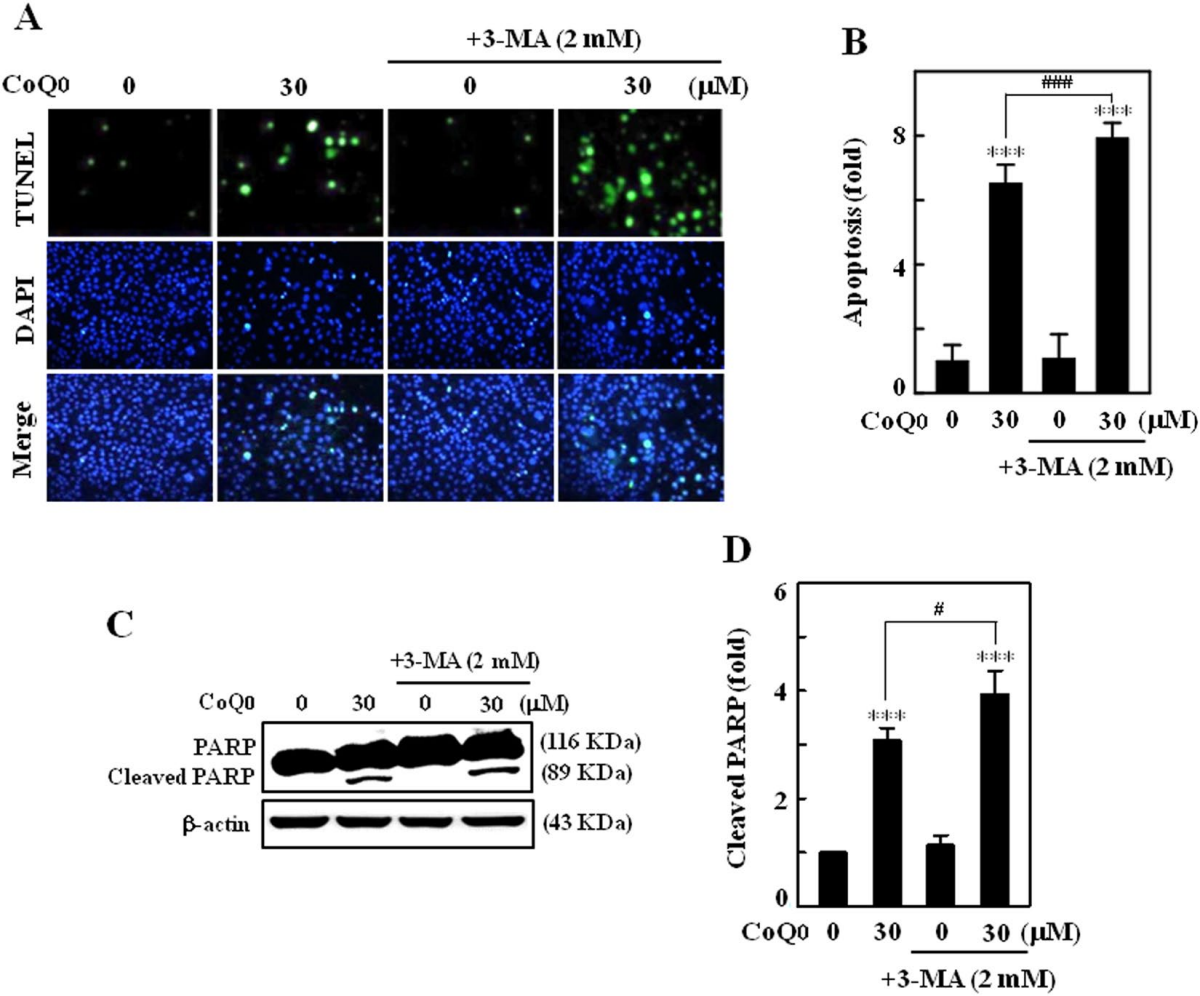
Inhibition of autophagy potentiated CoQ_0_-induced apoptosis in SKOV-3 cells. (**A–D**) Cells were pretreated with autophagy inhibitor (3-MA, 2 mM) for 1 h, and then incubated with CoQ_0_ (30 µM) for 24 h. **(A)** Induction of apoptosis (DNA fragmentation) was determined by TUNEL assay in the presence or absence of 3-MA. The green florescence indicates TUNEL-positive cells in microscopic fields (200 × magnification). **(B)** The fold of apoptotic cells was calculated by measuring the florescence intensity using commercially available software. **(C)** Cleavage of PARP with or without 3-MA pretreatment was determined by Western blot. **(C** Changes in protein intensities were quantified using commercially available software. Results expressed as mean ± SD of three independent assays (n = 3). Significant at ***p < 0.001 compared with untreated control, and significant at ^#^ *p* < 0.05; ^###^ *p* < 0.001 compared with CoQ_0_ alone treated cells.

To describe whether autophagy is able to influence CoQ_0_-induced apoptosis, SKOV-3 cells were pretreated with early autophagy inhibitor (3-MA, 2 mM), and occurrence of apoptotic DNA fragmentation and cleavage of PARP was measured. CoQ_0_ treatment (30 µM, 24 h) increased apoptotic DNA fragmentation (~6-fold) as evidenced by increased TUNEL-positive cells. Intriguingly, 3-MA pretreatment further intensified the apoptotic DNA fragmentation in SKOV-3 cells (Fig. 9A and B). 3-MA pretreatment enhanced the proteolytic cleavage of PARP that was prominent when compared with CoQ_0_ alone treatment (Fig. 9C). Evidence from our study (Fig. 3D, Fig. 9A–C) emphasizes that induction of cytoprotective autophagy may renders to delay or suppress the onset of CoQ_0_-induced apoptosis or SKOV-3 cell death.

### CoQ_0_ suppresses tumor growth in SKOV-3 xenografted nude mice

**Figure 10:**
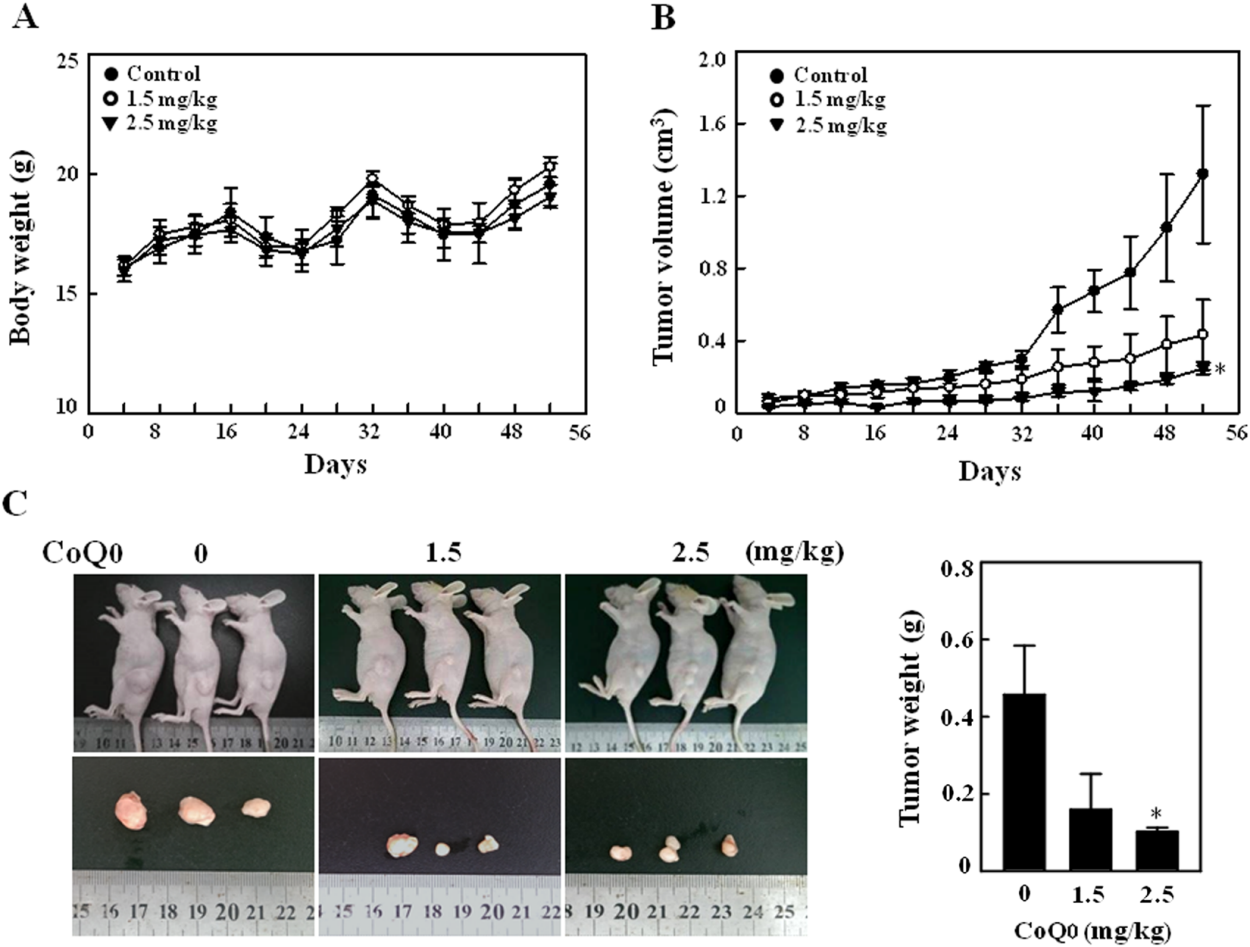
CoQ_0_ inhibits tumor growth in SKOV-3 xenografted nude mice. (A–C) Time-course effect of CoQ_0_ on growth of SKOV-3 xenografted nude mice was evaluated. Ovarian cancer cells (3 × 10^6^ cells) were subcutaneously implanted into the flanks of nude mice on day 0 and inoculated for 7 days. Mice were subsequently treated with CoQ_0_ (1.5 and 2.5 mg/kg) or vehicle (control) on every 4 days for 52 days. Changes in bodyweight **(A)** and tumor volume **(B)** were recorded for every 4 days, until 52 days. **(C)** On day 52, animals were photographed, and excised tumor tissue was weighed. Results expressed as mean ± SD of three independent assays (n = 3). Significant at *p < 0.05; compared with vehicle treated mice.

To examine the *in vivo* anti-tumor activity of CoQ_0_, we used nude mice and ovarian cancer SKOV-3 cells were subcutaneously xenografted into the mice, and then treated with CoQ_0_ (1.5 and 2.5 mg/kg) or vehicle for 52 days. During the experimental period no significant body weight loss was noticed among the groups, and all mice appeared healthy (Fig. 10A). Besides, no signs of the adverse effects or toxicity of CoQ_0_ was observed in nude mice, according to microscopic examination of individual organs (data not shown). The tumor volume was measured for every 4 days until 52 days, and found that both doses of CoQ_0_ (1.5 and 2.5 mg/kg) inhibited the tumor volume, higher dose being more effective in a time-course (Fig. 10B). Next, to assess CoQ_0_ effect on tumor weight, all mice including control were photographed, and excised xenografted tumor tissues were weighted. Consistent to the tumor volume, tumor weight was also considerably decreased with CoQ_0_ compared with control mice (Fig. 10C). These results revealed that CoQ_0_ suppressed the tumor growth in SKOV-3 xenografted nude mice.

### CoQ_0_ inhibits mitosis and promotes apoptosis in tumors of SKOV-3 xenografted mice

**Figure 11:**
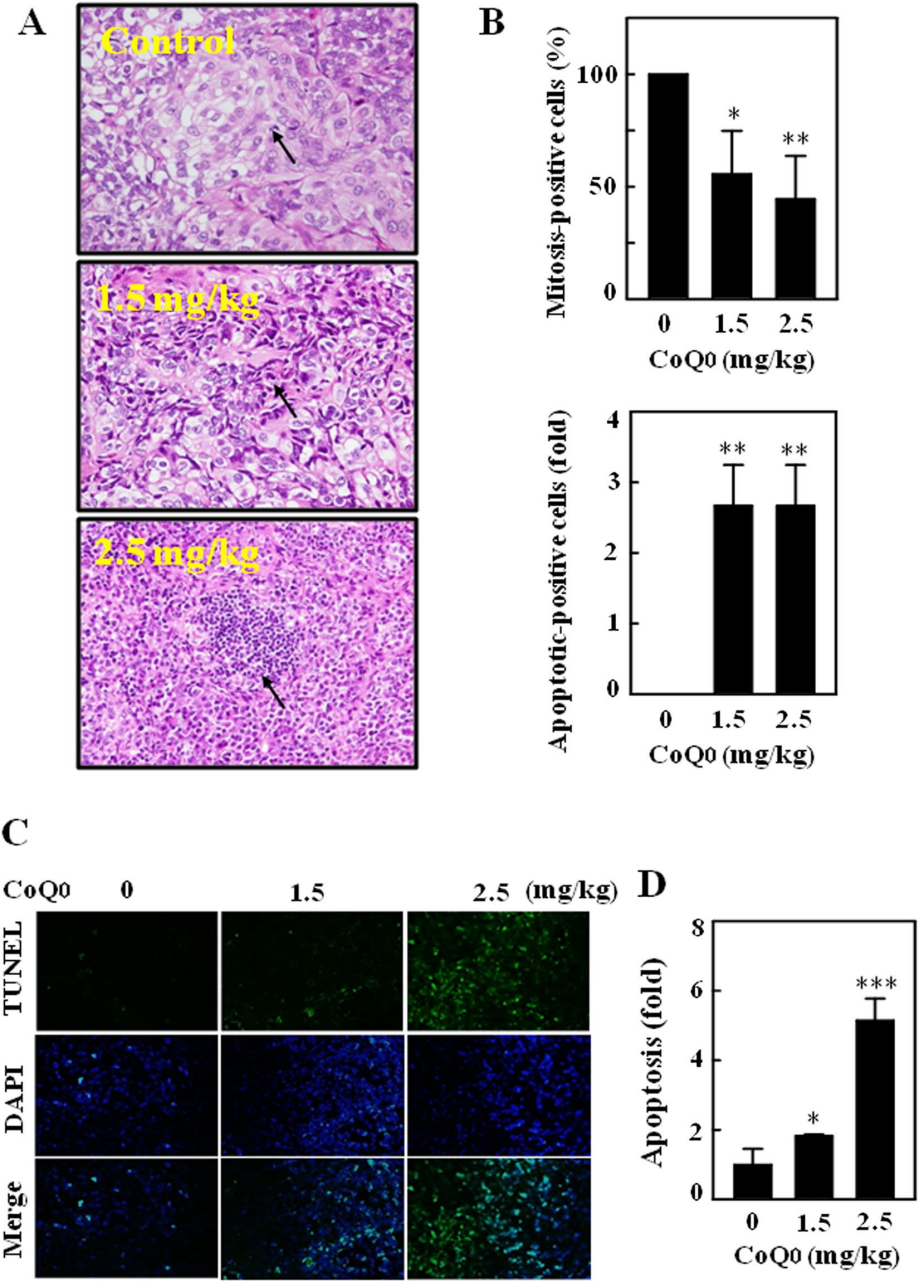
Histopathological analyses of mitosis and apoptosis in the tumors of SKOV-3 xenografted nude mice followed by CoQ_0_ treatment. (**A,B**) Histopathological staining was performed to detect the mitotic- and apoptotic-cells in SKOV-3 xenografted tumors following CoQ_0_ (1.5 and 2.5 mg/kg) treatment using light microscopy (20 × and 200 × magnification). Arrows indicate mitotic (tumor control) and pyknotic tumor cells (CoQ_0_). The number of mitotic-positive and apoptotic-positive cells in microscopic fields of 3 samples was averaged, and presented as histogram. (**C,D**) *In situ* detection of apoptosis was performed using TUNEL staining in tumor sections from control and experimental analogues treated with CoQ_0_ (1.5 and 2.5 mg/kg). The number of TUNEL-positive cells were quantified from 3 microscopic fields, and averaged. Results expressed as mean ± SD of three independent assays (n = 3). Significant at *p < 0.05; **p < 0.01; ***p < 0.001 compared with vehicle treated mice.

To delineate the reason why CoQ_0_ suppressed the tumor size and weight, we performed histopathological and TUNEL assays to examine the mitosis and apoptosis in tumor tissues. Microscopic images illustrated that the control tumor cells were large in size, round to oval in shape with predominant nucleoli. Tumor cells expressed high levels of cellular activity and mitotic figures, indicating proliferating cells. However, tumor sections of CoQ_0_-treated mice (1.5 and 2.5 mg/kg) showed smaller cells with shrunken, condensed and pyknotic nuclei, which indicates inactivity or regression of tumor cells (Fig. 11A). The quantified mitotic-positive cells that were abundant in control tumors were notably inhibited following CoQ_0_ treatment (Fig. 11B). Conversely, CoQ_0_ significantly promoted the apoptosis in tumors of xenografted mice (Fig. 11A and B), which was confirmed by a dose-dependent increase of apoptotic DNA fragmentation. Results from TUNEL assay showed CoQ_0_ treatment enhanced the TUNEL-positive cells compared with vehicle (Fig. 11C and D). Our evidence confirmed that the anti-tumor activity of CoQ_0_ in xenografted tumors is associated with decreased proliferation and increased apoptosis.

## Discussion

Owing to its potent anticancer/cytotoxic properties against human breast cancer^25^, and lung cancer cells^40^, we assume that CoQ_0_ could be a potential candidate to treat human ovarian carcinoma cells. For the first time, we have demonstrated that CoQ_0_, isolated from *Antrodia camphorata*, potently killed the ovarian SKOV-3 cancer cells and induced cell-cycle arrest *via* decreased cell-cycle regulatory proteins. CoQ_0_ triggered the intracellular ROS production, which then contributed to activate and propagate apoptotic signals. CoQ_0_-induced apoptosis was evidenced by increased apoptotic DNA fragmentation, higher number of early/late apoptotic cells and dysregulated Bax/Bcl-2 ratio. Both mitochondrial and ER-stress mediated apoptotic signals are implicated in execution of apoptosis. The blockade of ROS production by NAC treatment diminished pro-apoptotic signals and cell death. On the other hand, activation of autophagy by CoQ_0_ was evidenced by increased LC3-II accumulation, GFP-LC-3 punctuated patters and AVOs appearance, however not contributed for autophagic-cell death. Inhibition of apoptosis by Z-VAD-FMK reversed CoQ_0_-induced autophagy, while inhibition of autophagy by 3-MA/CQ exaggerated CoQ_0_-induced apoptotic signals. These findings explain that CoQ_0_-induced apoptosis evokes cytoprotective autophagy, which may counteract the CoQ_0_-induced pro-apoptotic signals and hinder the CoQ_0_ anticancer properties. Furthermore, CoQ_0_ treatment to SKOV-3 xenografted nude mice suppressed the tumor volume. Xenografted tumors after CoQ_0_ treatment represented by significantly decreased mitotic-cells and tremendously increased apoptotic-cells and DNA fragmentation. Taken together, our findings revealed that CoQ_0_ displays both *in vitro* and *in vivo* antitumor properties. CoQ_0_-induced ROS-mediates the apoptosis and activates cytoprotective/survival autophagy in human ovarian cancer cells.

Induction of apoptosis/autophagy, inhibition of cell survival and/or cell-cycle arrest by chemical substances or biological agents are the principal strategies in treatment of a variety of cancers, including ovarian carcinoma^19, 27, 41^. In this study, we demonstrated that CoQ_0_ treatment to ovarian cancer SKOV-3 cells produced potent cytotoxic effects, as indicated by a profound increase of cell death and aberrant morphological changes. Previous study reported that among several methoxy-substituted cyclic compounds, CoQ_0_ is the strongest cytotoxic analog that inhibited the growth of MDA-MB-231 and SKBr3 human breast cancer cells. The cytotoxicity of CoQ_0_ was associated with increased proportion of cells undergoing apoptosis, and this phenomenon was addressed by the induction of G_0_/G_1_ cell-cycle arrest in MDA-MB-231 cells and S-phase arrest in SKBr3 cells^25^. CoQ_0_ isolated from *Antrodia cinnamomea* submerged cultures has also reported to possess anticancer property by the decreased viability of A549, HepG2 and SW480 cancer cells, and increased ROS-mediated apoptosis^40^.

Previously, we have shown that treatment of SKOV-3 cells with fermented culture broth of *Antrodia camphorata* inhibited the proliferation, and caused G_2_/M cell-cycle arrest through the downregulation of cyclin D1, cyclin A and CDK1, and upregulation of p27 expressions^27^. The CoQ_0_ used in this study is a novel quinone derivative of *Antrodia camphorata*, which contain zero isoprenoid side chains. CoQ_0_-induced death of SKOV-3 cells in this study was also accompanied by the G_2_/M-phase cell-cycle arrest and decreased cell-cycle regulatory proteins (cyclin B1, CDK1, cyclin A and CDK2). Since overexpression of CDKs activity is often the cause of human cancer, their function is tightly regulated by cell-cycle inhibitors, such as the p21 and p27 Cip/Kip proteins. It has been stated that following anti-mitogenic signals or DNA damage, the proteins, p21 and p27 can bind to cyclin-CDK complexes to inhibit their catalytic activity and induce cell-cycle arrest^42^. In our study, the anti-mitogenic signals and apoptotic DNA damaging actions of CoQ_0_ perhaps activates p21 and p27 expressions, which then bind to cyclin-CDK complex to inhibit their expression in SKOV2 cells. Thus, suppression of cell-cycle promoting proteins and induction of cell-cycle arrest by CoQ_0_ may contribute to inhibit the ovarian cancer cell survival.

Aberrant production of ROS by oxidants or chemical substances play an important role in execution of apoptosis by activating the pro-apoptotic signals^33, 38^. Here we found that profoundly increased ROS production with CoQ_0_ was accompanied by an increased death of SKOV-3 cells, and blockade of ROS production by NAC treatment substantially diminished the CoQ_0_-induced cell death. Coenzyme Q exists in biomembranes act as an electron carrier and reversibly changes to either oxidized (CoQ), intermediate (CoQ^•−^) or reduced (CoQH_2_) form^24^. During conversion from one form to another, short chain CoQ^•−^ comes into contact with cytoplasm, auto-oxidation with O_2_ occurs and generates ROS^43^. Doughan *et al*., demonstrated that mitoquinone (MitoQ) may be the pro-oxidant and pro-apoptotic, because its quinone group can participate in redox cycling and superoxide radical production. MitoQ-induced ROS production appears to stimulate the activity of caspase-3 and endothelial cell apoptosis^8^. Ubiquinone 0 (Ub_0_) also known as CoQ_0_ reported to dramatic increase of ROS production in culture rat liver Clone-9 cells, which is prominent than the other analogs (Ub_5_ and Ub_10_). Particularly, Ub_0_ induced PTP opening in cancerous rat liver MH1C1 cells, and promoted PTP-dependent cell death^23^. Indeed, ROS accumulation can cause depolarization of mitochondrial membrane and results in increased release of mitochondrial proteins, including apoptosis-inducing factor (AIF) that certainly promote apoptosis^44, 45^. A study on human lung carcinoma cells showed that sodium selenite induced ROS-mediated intrinsic apoptosis and mitochondrial dysfunction was blocked by ROS inhibition^33^. Another recent study reported that CoQ_0_ provoked the ROS-mediated apoptosis of lung cancer cells, and increased apoptotic cells were noticeably inhibited with antioxidant pretreatment^40^. These results elucidate that the intracellular ROS are critically involved in the propagation of apoptosis in SKOV-3 cells.

In line with this, we demonstrated that CoQ_0_-induced apoptotic DNA fragmentation, PARP cleavage and early/late apoptosis were substantially reversed *via* ROS inhibition in SKOV-3 cells. CoQ_0_-iduced ROS-mediated apoptosis appears to be executed by mitochondrial- and ER stress-dependent signalling cascades. It has been described that the cleavage of procaspase-3 into active caspase-3 proceeds to fragmentation of PARP, and culminating the death of cell^10, 12^. PARP, an important nuclear enzyme involved in DNA repair is a substrate for activated caspase-3, and fragmentation of PARP is considered a hallmark of mitochondrial apoptosis^13^. Cleavage of procaspase-3 is an early event in apoptosis induced by chemotherapeutic agents, and activated caspase-3 also promotes DNA fragmentation^46^. Here, we found the marked increase of caspase-3 and PARP fragmentation in CoQ_0_-treated cells. Interestingly, pharmacological inhibition of caspase activation by Z-VAD-FMK or blockade of ROS production by NAC resulted in a diminution of CoQ_0_-iduced PARP fragmentation followed by a suppressed cell death. These findings support the occurrence of mitochondrial apoptosis in CoQ_0_-treated ovarian cancer cells. In fact, mitochondrial apoptosis is known to be stimulated by a wide range of cellular stresses, including overwhelming production of ROS, DNA damage and cytoskeletal disruption^12, 38^. CoQ_0_-iduced mitochondrial apoptosis was further addressed by the increased production of intracellular ROS levels and apoptotic DNA fragmentation in SKOV-3 cells.

ER stress, represented by an increased accumulation of unfolded proteins due to multiple stimuli, including oxidants, triggers a conserved series of signals to ameliorate the accumulation of unfolded proteins. However, if these signalling events are severe or protracted, they can induce cell death^11^. ER molecular chaperone, HSP70 and caspase-12 are said to be involved in the ER stress-specific apoptosis^11, 47^. We found increased expressions of caspase-12 and HSP-70 proteins in cells incubated with CoQ_0_, which implies the activation of ER stress-mediated apoptosis. Caspase-12 is predominantly localized to the ER, and specifically activated by the apoptotic signals with an ER stress component^47^. The bile salt sodium deoxycholate, an apoptosis inducer was found to activate the HSP-70 in liver cells along with other genes involved in ER stress and DNA damage. This study suggested that activated chaperone protein HSP-70 may be an additional stress response due to malfolding of proteins in ER^48^.

Our findings further demonstrated that CoQ_0_ induced early/late apoptotic death of SKOV-3 cells. Along with aberrant morphological changes, results from Annexin-V/PI assay showed the greater number of early (Annexin-V^+ve^/PI^−ve^) and late (Annexin-V^+ve^/PI^+ve^) apoptotic cells following CoQ_0_ treatment. However, this phenomenon was not observed in NAC pretreated cells, which indicates the involvement of ROS in apoptosis. Treatment of HL60 cells with different CoQ structures (CoQ_1_, CoQ_2_ and CoQ_4_) reported to inhibit the proliferation, initial S-phase arrest followed by G_0_/G_1_-phase cell-cycle arrest at later time points, and induction of apoptosis^49^. A recent study by Chung and colleagues showed that CoQ_0_ enhanced both early and late apoptosis in human lung cancer cells, and antioxidant pretreatment diminished the apoptotic-cell death^40^. Several studies demonstrated that quinones or certain methoxy-containing analogs of CoQ with structural similarities to CoQ_0_ are able to produce cytotoxic effects on human cancer cells by the induction of apoptosis^24, 49^. A study carried-out using different CoQ analogs reported the increased apoptosis, DNA fragmentation and caspase-3 activation with CoQ_2_ and CoQ_4_, but not with CoQ_6_ or CoQ_10_ in HL60 human leukemia cells^49^. These findings imply that the anticancer or pro-apoptotic properties of CoQ analogs may be varied based on the length of isoprenyl side chain, and position of the methoxy-substitutions on the quinone nucleus. Irrespective of cell lines, CoQ_0_ that contains zero isoprenoid units inhibit ovarian cancer cell growth and induce early/late apoptosis through the ROS signals.

On the other hand, emerging evidence shows that autophagy constitutes a potential target for cancer therapy, and induction of autophagy in response to therapeutics can be viewed as a cell-death or cell-survival phenomenon that contributes to the anticancer efficacy^19^. For the first time, we demonstrated the novel function of CoQ_0_, that is induction of autophagy in the ovarian cancer cells. This phenomenon was evidenced by the increased conversion of LC3-I to LC3-II (membrane bound), multiplied AVOs formation and elevated Beclin-1 expression following CoQ_0_ treatment. Induction of autophagy by CoQ_0_ was further confirmed by the confocal images illustrating the abundance of green GFP-LC3 puncta. The increased appearance of AVOs, a hallmark of autophagy is associated with the increased accumulation of lipidated LC3 in cells^15^. CoQ_0_-induced higher numbers of AVOs and corresponding increased distribution of GFP-LC3-II punctuates in SKOV-3 cells are explaining the recruitment of LC3-II to autophagosomes. Antroquinonol, a ubiquinone derivative of *Antrodia camphorata* has been reported to increase the LC3-II accumulation and a certain degree of autophagic-death of pancreatic carcinoma cells^30^. Since mTOR signalling is considered a key negative regulator of autophagy^50^, we determined the CoQ_0_ effect on phosphorylated mTOR levels, and found severely inhibited p-mTOR (S2448) levels in SKOV-3 cells. This was accompanied by a simultaneous decrease of p-AKT levels and increase of Beclin-1/Bcl-2 ratio, which denotes activation of autophagy in SKOV-3 cells. It has been claimed that excessive production of ROS can act as intracellular messengers to trigger autophagy^39^. To address whether CoQ_0_-induced ROS involved in activation of autophagy in SKOV-3 cells, we detected the AVOs formation in the presence and absence of NAC. It is interesting to note that inhibition of ROS production by NAC is unable to suppress the CoQ_0_-induced AVOs formation, which indicates CoQ_0_ activated autophagy is ROS-independent. Taken together, our results clearly explained that CoQ_0_-induced ROS are involved in activation of the pro-apoptotic signals, but not autophagy in ovarian cancer cells.

Another most important finding of this study is that CoQ_0_ activated autophagy in SKOV-3 cells didn’t contribute for cell death; instead it might be a cytoprotective mechanism. There are confirmatory evidence explaining that induction of apoptotic cancer cell death by chemical substances is accompanied by a protective autophagy^18, 21, 33^. Activation of autophagic pathway in response to target therapies or metabolic stress reportedly contributes to the survival of formed tumors, which might mediate resistance to the anticancer therapies^17, 51^. Of note, without addressing the role of autophagy, few studies enlightened the cytotoxicity of CoQ_0_ against human lung cancer cells^40^ and breast cancer cells^25^ *via* induction of apoptotic-cell death. Yet it is unclear, whether CoQ_0_-induced autophagy contributes to survival or death of ovarian cancer cells. First time, we demonstrated that inhibition of CoQ_0_-induced autophagy by 3-MA or CQ didn’t suppress the CoQ_0_-induced cell death. These evidence explain that CoQ_0_-induced autophagy is not involved in death of ovarian cancer cells, perhaps play a self-protective role against the cytotoxic effects of CoQ_0_. However, it can’t be ruled out that activation of autophagy by anticancer drugs/oxidants act as an alternative pathway for cellular death^17^. Although autophagy role is controversial in cancer cells, our findings suggest that autophagy may serves as an additional target for adjuvant anticancer therapy, and inhibition of such autophagy could further strengthen the therapeutic effectiveness of CoQ_0_ treatment.

The interaction between autophagy protein (Beclin-1) and anti/pro-apoptotic proteins (Bcl-2/Bcl-XL) is complex, and represents a potential important point of convergence of the apoptotic and autophagic machinery^20, 52^. It has been shown that Beclin-1 cannot neutralize the Bcl-2 function, but Bcl-2 family proteins suppress the pro-autophagic function of Beclin-1^14^. In our study, CoQ_0_ downregulated the Bcl-2 and upregulated the Beclin-1 that indicates activation of respective apoptosis and early autophagy mechanisms in SKOV-3 cells. Similar to our studies, anticancer properties of Sulforaphane were represented by the decreased Bcl-2 levels and increased caspase-3 activation in human breast cancer cells^15^. Activated caspase-3 can cleave Beclin-1 protein at position 125 and 149. The truncated Beclin-1, which in turn disrupt its interaction with Bcl-2 then allows the release of pro-apoptotic molecules from the Bcl-2/Bcl-xL complex to initiate intrinsic apoptosis^53^. In addition, caspase-mediated cleavage of Beclin-1 promote the crosstalk between apoptosis and autophagy^14^. CoQ_0_-induced elevated Beclin-1/Bcl-2 ratio and Bax/Bcl-2 ratio in ovarian cancer cells suggest that CoQ_0_ promotes cell death *via* pro-apoptotic signals.

HER-2/*neu* is a proto-oncoprotein belongs to the family of epidermal growth factor, overexpressed in various human cancers, including ovarian carcinoma. Overexpression of HER-2/*neu* is associated with the high risk of treatment due to highly metastatic ability of cells and resistant to drug treatments^54^. We demonstrated that CoQ_0_ treatment to ovarian cancer cells time-dependently inhibited the HER-2/*neu* expression. The decreased HER-2/*neu* phosphorylation was accompanied by a substantial reduction of AKT and mTOR phosphorylations. It has been shown that activation of HER-2 leads to autophosphorylation of the C-terminal tyrosines of the receptor, and implicated in regulation of cell proliferation and inhibition of apoptosis^55^. Therefore, inhibition of p-HER-2 by CoQ_0_ indicates decreased proliferation and increased apoptosis of SKOV-3 cells. In our previous study, we have shown that *Antrodia camphorata* that contains CoQ_0_ significantly decreased the basal tyrosine kinase phosphorylation and activation of HER-2/*neu* receptors in HER-2/*neu*-overexpressed SKOV-3 cells^27^. A recent study showed that downregulation of HER-2 by Alo-emodin in breast cancer cells resulted in decreased tumor initiation, cell migration and invasion^9^. Since overexpression of HER-2 has been linked with activation of AKT/mTOR signals that are involved in tumor biology^27, 37^, the decreased AKT/mTOR phosphorylation with CoQ_0_ perhaps contributes to effective management of the ovarian cancer progression.

Apoptosis and autophagy shares the functions of several common regulators, and the crosstalk between apoptosis and autophagy decides the fate of cell in response to cellular stress^34^. Regardless of their complex interrelationship, autophagy usually associated with cell survival, whereas apoptosis is invariably contributes to cell death^15, 56^. Accumulating evidence suggests that under certain stress circumstances, autophagy is known to act as a partner or a promoter of apoptosis. Conversely, activation of autophagy may function to prevent the onset of apoptosis, and apoptosis can also activate autophagy^21, 34, 57^. It has been shown that oridonin treated L929 cells exhibited both autophagy and apoptosis, where inhibition of autophagy increased the apoptotic-cell death, which means autophagy has an anti-apoptotic function^56^. Owing to the complex interactions between apoptosis and autophagy, it is necessary for careful monitoring of the key regulatory molecules to understand the cell death phenomenon induced by chemical substances. We demonstrated that CoQ_0_-induced apoptosis contributes to death of ovarian cancer cells, rather than autophagy. Chemical inhibition of apoptosis diminished the CoQ_0_-induced cell death by inhibition of PARP cleavage and DNA fragmentation.

Since occurrence of apoptosis always means to cell death^12^, the reported autophagy in CoQ_0_-treated cancer cells does not necessarily indicates autophagic-cell death. It is worth to note that inhibition of autophagy is unable to diminish the CoQ_0_-induced cell death; despite intensified the apoptotic signals. On the other hand, inhibition of apoptosis is accompanied by a substantial diminution of CoQ_0_-induced autophagy (decreased LC3-II accumulation and AVOs formation). Cellular stresses, such as ER-stress and mitochondrial dysfunction are reported to activate the autophagy^57^. Likewise, ginsenosides F2 induces mitochondrial dysfunction and apoptotic death of breast cancer cells was accompanied by autophagy, where inhibition of autophagy enhanced the F2-induced cell death that reveals the protective role of autophagy^21^. Thus, CoQ_0_-induced ER-stress and increased release of mitochondrial apoptotic proteins may be resulted in activation of the autophagy mechanism to remove the damaged organelles. These novel findings addressed that CoQ_0_-induced autophagy may not participate in cell death, perhaps served as a survival mechanism against cytotoxicity and cellular stress.

To further strengthen the anticancer properties of CoQ_0_, we performed the *in vivo* studies on SKOV-3 xenografted nude mice by treating with CoQ_0_. We found that treatment of CoQ_0_ to xenografted nude mice significantly decreased the tumor volume. This antitumor activity appears to be associated with the inhibition of mitotic cells and a dramatic increase of apoptotic cells/DNA fragmentation in CoQ_0_-treated tumors. A study by Zhang and colleagues demonstrated that treatment with EB1089, an analog of vitamin D completely suppressed the growth of tumors in ovarian cancer xenografted nude mice. In addition, fewer mitotic figures and increased TUNEL-positive apoptotic cells were also observed in the tumor sections of EB1089 treated nude mice^58^. The decreased mitotic-positive cells in the tumors of *Antrodia salmonea* treated HL60 xenografted nude mice indicating the decreased cell proliferation, which may result in decreased tumor volume in nude mice^59^. A recent study suggests that suppression of epithelial ovarian cancer cell invasion into the omentum by EB1089 may help to improve the survival of patients with advanced ovarian cancer^3^. CoQ_0_ treatment to xenografted nude mice contributed to decrease the cancer cell proliferation and increased apoptosis. These *in vivo* findings confirmed the potent antitumor properties of CoQ_0_ against ovarian cancer that are consistent with *in vitro* anticancer properties. Take into consideration of *in vitro* evidence, CoQ_0_-induced ROS possibly involved in propagation of apoptosis in xenografted tumors.

## Conclusions

For the first time, our findings demonstrated that CoQ_0_ induced apoptotic-death of human ovarian cancer cells through the increased production of ROS. The blockade of ROS production by NAC suppressed CoQ_0_-induced apoptotic signals and diminished cell death, but not autophagy. Chemical inhibition of apoptosis substantially retarded the CoQ_0_-induced autophagy and cell death. However, inhibition of autophagy was unable to suppress the CoQ_0_-induced apoptosis, instead accelerated the pro-apoptotic signals. Our experimental evidence provides insights into the complex relation between apoptosis and autophagy induced by CoQ_0_ in SKOV-3 cells. We further demonstrated the *in vivo* antitumor properties of CoQ_0_ in SKOV-3 xenografted nude mice. We found that CoQ_0_ treatment substantially inhibited the tumor volume through decreased mitotic-cells and increased apoptotic-cells in tumors. These findings emphasize that CoQ_0_ revealed potent anticancer properties through ROS-mediated apoptosis against human ovarian carcinoma cells. The inhibition of cytoprotective autophagy or further amplifying of intracellular ROS production could be the potential strategies to improve the effeteness of cancer treatment.

## Materials and Methods

### Chemicals and reagents

Dulbecco’s Modified Eagle’s medium (DMEM), nutrient mixture F-12, fetal bovine serum (FBS), glutamine and penicillin/streptomycin were obtained from GIBCO BRL (Grand Island, NY, USA). 3-(4,5-Dimethylthiazol-2-yl)-2,5-diphenyltetrazolium bromide (MTT), *N*-acetylcysteine (NAC), *p*-iodonitrotetrazolium violet, fluorescein isothiocyanate (FITC), propidium iodide (PI), acridine orange (AO), 3-Methyladenine (3-MA), chloroquine (CQ) and 2′,7′-dihydrofluorescein-dictate (DCFH_2_-DA) were purchased from Sigma-Aldrich Chemical Co. (St. Louis, MO, USA). Antibodies against cyclin B1, CDK1, cyclin A, CDK2, caspase-3, Bcl-2, Bax, and β-actin were purchased from Santa Cruz Biotechnology, Inc. (Heidelberg, Germany). Antibodies against LC3-I/II, PARP, p-HER-2/*neu*, p-AKT, p-mTOR, and Bcelin-1 were obtained from Cell Signalling Technology, Inc. (Danvers, MA, USA). Antibody against GFP was purchased from Gene Tex, Inc. (Irvine, CA, USA). Antibodies against HS-70 and Caspase-12 were purchased from BD Transduction Laboratories (Hayward, CA, USA) and Millipore Corporation (Billerica, MA, USA) respectively. 4′,6-Diamidino-2-phenylindole dihydrochloride (DAPI) was purchased from Calbiochem (La Jolla, CA, USA). Z-Val-Ala-Asp-fluoromethylketone (Z-VAD-FMK) was obtained from Calbiochem (San Diego, CA, USA). All other chemicals were reagent grade or HPLC grade and were supplied by either Merck & Co., Inc. (Darmstadt, Germany) or by Sigma-Aldrich.

### Preparation of fermented culture broth of *Antrodia camphorata* from submerged cultures

The *Antrodia camphorata* culture was inoculated onto potato dextrose agar and incubated at 30 °C for 15–20 days. The whole colony was subsequently added to a flask containing 50 mL sterile water. After homogenization, the fragmented mycelial suspension was used as an inoculum. The seed culture was prepared in a 20-L fermenter (BioTop Process & Eqipment, Taiwan) agitated at 150 rpm with an aeration rate of 0.2 vvm at 30 °C. A five-day culture of 15 L mycelium inoculum was inoculated into a 250 L agitated fermenter (BioTop). The fermentation conditions were the same as those used for the seed fermentation, but the aeration rate was 0.075 vvm. The fermentation product was harvested at hour 331 and poured through a non-woven fabric on a 20-mesh sieve to separate the deep-red fermented culture broth and the mycelia; the culture broth was then centrifuged at 3000 × *g* for 10 min followed by passage through a 0.22-μm filter. The culture broth was concentrated under vacuum and freeze-dried to a powder. The yield of dry matter from the culture broth was 18.4 g/L. The experiments were performed with 2–4 different batches of *Antrodia camphorata* fermented culture^60^.

### Isolation and characterization of CoQ_0_ from *Antrodia camphorata*

The HPLC profile of the fermented culture broth of *Antrodia camphorata* was performed using a RP-18 column [COSMOSIL, 5C_18_-AR-II, Waters, 4.6 × 250 mm] at a flow rate of 1.0 mL/min, detected at UV 254 and 220 nm. Standard solution of the fermented culture broth from *Antrodia camphorata* was prepared by dissolving it in water (5.0 mg/mL), filtered through 0.22 μm membrane filter and applied to HPLC analysis. The mobile phase consisted of (A) acetonitrile and (B) 0.05% trifluoroacetic acid (TFA) (v/v) using a gradient elution of 5–60% A at 5–40 min. The flow rate was maintained as 1.0 mL/min and aliquots of 20 μL were injected. According to our previous results of HPLC analysis, the amounts of CoQ_0_ (Fig. 1A) in the fermented culture broth of *Antrodia camphorata* were 17.3% (254 nm) and 13.5% (220 nm), respectively^60^. The purity of CoQ_0_ is ≥98%, which is similar to commercially available CoQ_0_.

### Cell culture and treatment

Human ovarian cancer (SKOV-3) cells were obtained from the American Type Culture Collection (ATCC, Manassas, VA, USA). Two epithelial ovarian cell lines (A2780 and CP70) were kindly provided by Dr Cheng-I Leen (National Chung Cheng University, Taiwan). Human ovarian surface epithelial (IOSE) cells were kindly provided by Dr Michael Chan (National Chung-Cheng University, Taiwan). Cells were cultured in DMEM/F12 supplemented with 10% heat-inactivated FBS, 2 mM glutamine and 1% penicillin- streptomycin-neomycin at 37 °C in a humidified incubator with 5% CO_2_. Cultures were harvested and monitored for changes in cell number by counting cell suspensions using a hemocytometer (Marienfeld, Germany). Cell morphology was examined using phase contrast microscopy (200 × magnification). Cells were treated with increasing concentrations of CoQ_0_ (0–40 µM), the incubation time was varied depending on the assay. Wherever applicable, cells were pretreated with various pharmacological inhibitors, including NAC (2 mM), Z-VAD-FMK (20 μM), 3-MA (2 mM) or CQ (10 μM) for 1 h, and then incubated with indicated concentration of CoQ_0_ for 24 h.

### Assessment of cell viability by MTT assay

The effect of CoQ_0_ on viability of human ovarian carcinoma **(**SKOV-3, A2870 and A2870/CP-70) and human ovarian surface epithelial (IOSE) cells was determined by the MTT colorimetric assay. Briefly, cells (2.5 × 10^4^ cells/well in 24-well plates) were treated with different concentrations of CoQ_0_ (0–40 µM) for 24 h. After CoQ_0_ treatment, 400 μL 0.5 mg/mL MTT in PBS was added to each well. After incubation at 37 °C for 2 h, an equal volume of DMSO (400 μL) was added to dissolve the MTT formazan crystals, and the absorbance was measured at 570 nm (A_570_) using an ELISA microplate reader (µ-Quant, Winoosky, VT, USA). The percentage (%) of cell viability was calculated as: (A_570_ of treated cells/A_570_ of untreated cells) × 100. The assay was performed in triplicate at each concentration.

### Cell-cycle analysis

Cellular DNA content was determined by flow cytometry using the propidium iodide (PI)-labeling method as described previously^31^. Briefly, cells were seeded at a density of 4 × 10^5^ cells/dish in 10 cm dishes, and the cell-cycle was synchronized by the addition of double thymidine (3 mM) for 16 h. Cell-cycle-synchronized cells were then washed with PBS and re-stimulated to enter the G1 phase together by the addition of fresh medium, which also contained various concentrations of CoQ_0_ (0–30 µM). Cells were harvested at 24 h, and the cell-cycle analysis was performed using a FAC-Scan cytometry assay kit (BD Biosciences, San Jose, CA, USA) equipped with a single argon ion laser (488 nm). The DNA content of 1 × 10^4^ cells/analysis was monitored using the FACS Calibur system. Cell-cycle profiles were analyzed with ModFit software (Verity Software House, Topsham, ME, USA).

### Protein isolation and immunoblotting

Cells (1 × 10^6^cells/10-cm dish) were incubated with CoQ_0_ (0–30 µM) for the indicated time periods. After incubation, cells were harvested, pooled, washed once with PBS and suspended in 89 μL of lysis buffer (10 mM Tris-HCl, pH 8, 32 mM sucrose, 1% Triton X-100, 5 mM EDTA, 2 mM DTT and 1 mM phenylmethyl sulfonyflouride). The cell lysates were maintained on ice for 30 min and then centrifuged at 12000 rpm for 30 min at 4 °C. Total protein content was determined using Bio-Rad protein assay reagent (Bio-Rad, Hercules, CA, USA) with bovine serum albumin as a standard. The protein extracts were mixed with sample buffer (62 mM Tris-HCl, 2% SDS, 10% glycerol, and 5% β-mercaptoethanol), and the mixture was boiled at 97 °C for 5 min. Equal amounts (50 μg) of denatured protein samples were separated by 8–18% SDS-PAGE and then transferred onto polyvinylidene difluoride (PVDF) membranes overnight. The membranes were blocked with 5% non-fat dried milk in PBS containing 1% Tween-20 for 1 h at room temperature, followed by incubation with primary antibodies for overnight. The membranes were then incubated with either a horseradish peroxidase (HRP)-conjugated anti-rabbit or anti-mouse antibodies for 2 h prior to development using a Chemiluminescent substrate (Millipore, Billerica, MA, USA). The changes in protein intensities were digitized using the ImageQuant™ LAS 4000 mini (Fujifilm). Densitometric analyses were performed using commercially available quantitative software (AlphaEase, Genetic Technology Inc. Miami, FL, USA). Each assay was performed in triplicate, with control representing 1 fold, and changes in protein intensities were presented as histograms.

### Measurement of intracellular ROS production

Intracellular ROS accumulation was detected by fluorescence microscopy using the cell-permeable fluorogenic probe DCFH_2_-DA. Cells (2.5 × 10^4^ cells/mL) were cultured in DMEM/F12 medium that had been supplemented with 10% FBS, and the culture medium was replaced when the cells had reached 80% confluence. To evaluate ROS generation in a time-dependent manner, the cells were treated with CoQ_0_ (0–30 µM) for 0–30 min. Then culture supernatants were removed, and incubated with non-fluorescent DCFH_2_-DA (10 μM) in fresh medium at 37 °C for 30 min. Intracellular ROS production was quantified by measuring the intracellular accumulation of dichlorofluorescein (DCF), which is caused by the oxidation of DCFH_2_. The DCF-stained cells were captured using fluorescence microscope (200 × magnification) (Olympus, Center Valley, PA, USA). The fluorescence intensity was quantified using analysis LS 5.0 soft image solution (Olympus Imaging America Inc.,). The percentage of fluorescence intensity (ROS generation) was compared with untreated control cells, which were arbitrarily assigned a value of 100%.

### Detection and quantification of acidic vesicular organelles (AVOs) formation

Formation of AVOs was detected using AO stain in SKOV3 cells treated with various concentrations of CoQ_0_ (0–30 µM) for 24 h. Briefly, after designated treatments cells were washed with PBS twice, followed by staining with AO (1 μg/mL) and dilution in PBS containing 5% FBS for 15 min. After staining, cells were washed with PBS and covered with PBS containing 5% FBS. The cells were observed under a red filter fluorescence microscope and formation of AVOs in cells was visualized at 200 × magnification. AO is a lysosomotropic metachromatic and weak base membrane-permeant fluorescent dye, whose fluorescence emission is concentration dependent, from red at high concentrations (in lysosomes) to green at low concentrations (in the cytosol), with yellow as intermediate in some conditions^61^.

### GFP-LC3 plasmid transfection and detection of GFP-LC3 dot formation in cells

LC3 cDNA was a kind gift from Dr. Jiunn- Liang Ko (Chung-Shan Medical University, Taiwan). GFP-LC3 fusion protein was used to make the autophagosomes visible in cells. The cells were seeded onto coverslips placed onto a 6-well plate (1.8 × 10^5^ cells/well). After overnight culture, cells were transfected with 2.5 μg GFP-LC3 expressing plasmid in each well of a 6-well plate using Lipofectamine (Invitrogen, Carlsbad, CA, USA) and incubated for 24 h. The medium was removed and fresh medium containing CoQ_0_ (0–30 µM) was added to the wells. At the end of CoQ_0_ treatment (24 h), cells were washed twice with PBS, and expression of GFP-LC3 dots in cells were detected by a laser scanning confocal microscope at 200 × magnification.

### Determination of apoptotic cells by Annexin-V/PI staining

Double staining for Annexin-V-FITC and PI (propidium iodide) was performed to estimate the apoptotic rate of SKOV-3 cells. Briefly, cells were incubated with CoQ_0_ (0–30 µM) for 24 h, trypsinized, washed twice with PBS, and centrifuged at 1000 rpm for 5 min. Then, cells (1 × 10^6^cells/10-cm dish) were suspended in binding buffer (500 μL) and double-stained with Annexin-V-FITC and PI for 15 min at room temperature. Then the result green (FITC) and red (PI) fluorescence of each sample was quantitatively analyzed by FACS Caliber flow cytometer (Becton Dickinson, San Jose, CA, USA) and Cell Quest software. The obtained results were interpreted as follows: (Q3) cells negative for both PI and Annexin-V-FITC staining were considered normal live cells. (Q4) PI-negative, Annexin-V- FITC-positive stained cells were considered in early apoptosis. (Q2) PI-positive, Annexin-V-FITC-positive stained cells were considered in late apoptosis. (Q1) PI-positive, Annexin-V-FITC-negative stained cells were considered in necrosis.

### Apoptotic DNA fragmentation by TUNEL assay

DNA fragmentation in cells was measured using commercially available TUNEL assay kit (Calbiochem, San Diego, CA, USA). After treatment with designated inhibitors (1 h) and CoQ_0_ (0–30 µM, 24 h), apoptotic cells (2 × 10^4^ cells/well in 8-well chamber) were harvested, fixed with 4% formaldehyde and mounted on glass slides. Apoptosis was detected by labeling the 3′-OH ends of fragmented DNA with biotin-dNTP using DNA I klenow fragment at 37 °C for 1.5 h. The slides were then incubated with horseradish peroxidase-conjugated streptavidin, followed by incubation with 3,3′-diaminobenzidine and H_2_O_2_. The fragmented DNA was identified by their fluorescence nuclei under a fluorescence microscope (200 × magnification). The green fluorescence intensity was quantified using a squared section of fluorescence-stained cells with analysis LS 5.0 soft image solution (Olympus Imaging America Inc., PA, USA). The percentage of fluorescence intensity is directly proportional to the percentage of apoptotic cells, compared to untreated control cells, which were arbitrarily assigned a value of 100%.

### Animals

Female athymic nude mice (BALB/*c-nu*), 5–6 weeks of age, were purchased from The National Laboratory Animal Center (Taipei, Taiwan) and were maintained in caged housing in a specifically designed pathogen-free isolation facility with a 12 h/12 h light/dark cycle. The mice had free access to rodent chow (Oriental Yeast Co Ltd., Tokyo, Japan) and water *ad libitum*. All animal experiments were strictly followed “The Guidelines for the Care and Use of Laboratory Animals” published by the Chinese Society of Animal Science, Taiwan. The entire animal study protocols were reviewed and approved by the Institutional Animal Care and Use Committee (IACUC) of China Medical University, Taichung, Taiwan.

### Tumor cell inoculation and CoQ_0_ treatment

A total of 9 mice were randomly divided into three groups containing three animals in each group. SKOV3 cells (3 × 10^6^ cells, 100 μL) were mixed in a 100 μL matrix gel and then injected subcutaneously in a volume of 200 μL into the right hind flanks of nude mice. The experiments were performed using cells that had been passaged fewer than 20 times. After cell inoculation for 7 days, the treatment groups received CoQ_0_ (1.5 and 2.5 mg/kg b.w.) in a volume of 100 μL *via* intraperitoneal injection every 4 days for 52 days. The control group received the vehicle (PBS) only. CoQ_0_ was dissolved in PBS (containing 1% DMSO) and injections were scheduled in the morning between 10 AM and 11 AM throughout the study. To monitor drug toxicity, the body weight of each animal was measured for every 4 days. Tumor volume in mice was compared with caliper measurements of tumor length, width and depth, and then calculated every 4 days using the formula: length × width^2^ × 1/2. On day 52, all mice were sacrificed and tumor tissues were removed and weighed. A veterinary pathologist examined the mouse organs, including the liver, lungs and kidneys.

### Histopathological analyses

The biopsied tumor tissues were embedded in paraffin and cut into 3 mm thick sections, placed in plastic cassettes and immersed in neutral buffered formalin for 24 h. The fixed tissues were processed routinely and then embedded in paraffin, sectioned, deparaffinised, and rehydrated using standard techniques. The extent to which treatment shrunk tumor cells was evaluated by assessing the mitotic cell division in xenografted tumor sections using hematoxylin and eosin (H & E) staining for light microscopy. Then the number of mitotic cells or apoptotic cells in microscopic fields were averaged and presented as histogram. TUNEL apoptosis in the sections of CoQ_0_ or vehicle treated tumors was measured using *in situ* cell death detection kit (Roche, Mannheim, Germany).

### Statistical analyses

Data from *in vitro* experiments were presented as mean and standard deviation (mean ± SD). For *in vivo* experiments, mean data values are presented with standard error (mean ± SE). Data from both studies were analyzed using analysis of variance followed by Dunnett’s test for pair-wise comparison. Statistical significance was defined as *p < 0.05, **p < 0.01, ***p < 0.001 compared to untreated control cells, and significant at ^#^ *p* < 0.05; ^##^ *p* < 0.01; ^###^ *p* < 0.001 compared to CoQ_0_ alone treated cells.

## Electronic supplementary material

Supplementary Information
