# Supplementary material for: Antitumor properties of Coenzyme Q0 against human ovarian carcinoma cells via induction of ROS-mediated apoptosis and cytoprotective autophagy
Source: Sci Rep. 2017 Aug 14;7:8062. doi: 10.1038/s41598-017-08659-7 (PMC5556069; doi:10.1038/s41598-017-08659-7)
Supplement: Supplementary file 1 — Supplementary Information [file 41598_2017_8659_MOESM1_ESM.pdf]

SREP-17-17838A_Supplemenrary Information

Antitumor properties of Coenzyme Q_0 against human ovarian carcinoma cells *via* induction of ROS-mediated apoptosis and cytoprotective autophagy

You-Cheng Hseu[a,b], Tai-Jung Tsai[c], Mallikarjuna Korivi[c], Jer-Yuh Liu[d], Hui-Jye Chen[e], Chung-Ming Lin[f], Yi-Chun Shen[c], Hsin-Ling Yang[c,*]

[a]Department of Cosmeceutics, College of Biopharmaceutical and Food Sciences, China Medical University, Taichung 40402, Taiwan
[b]Department of Health and Nutrition Biotechnology, Asia University, Taichung 41354, Taiwan
[c]Institute of Nutrition, College of Biopharmaceutical and Food Sciences, China Medical University, Taichung 40402, Taiwan
[d]Graduate Institute of Cancer Biology, China Medical University, Taichung 40402, Taiwan
[e]Graduate Institute of Basic Medical Science, China Medical University, Taichung 402, Taiwan
[f]Department of Biotechnology, Ming Chuan University, Taoyuan 333, Taiwan

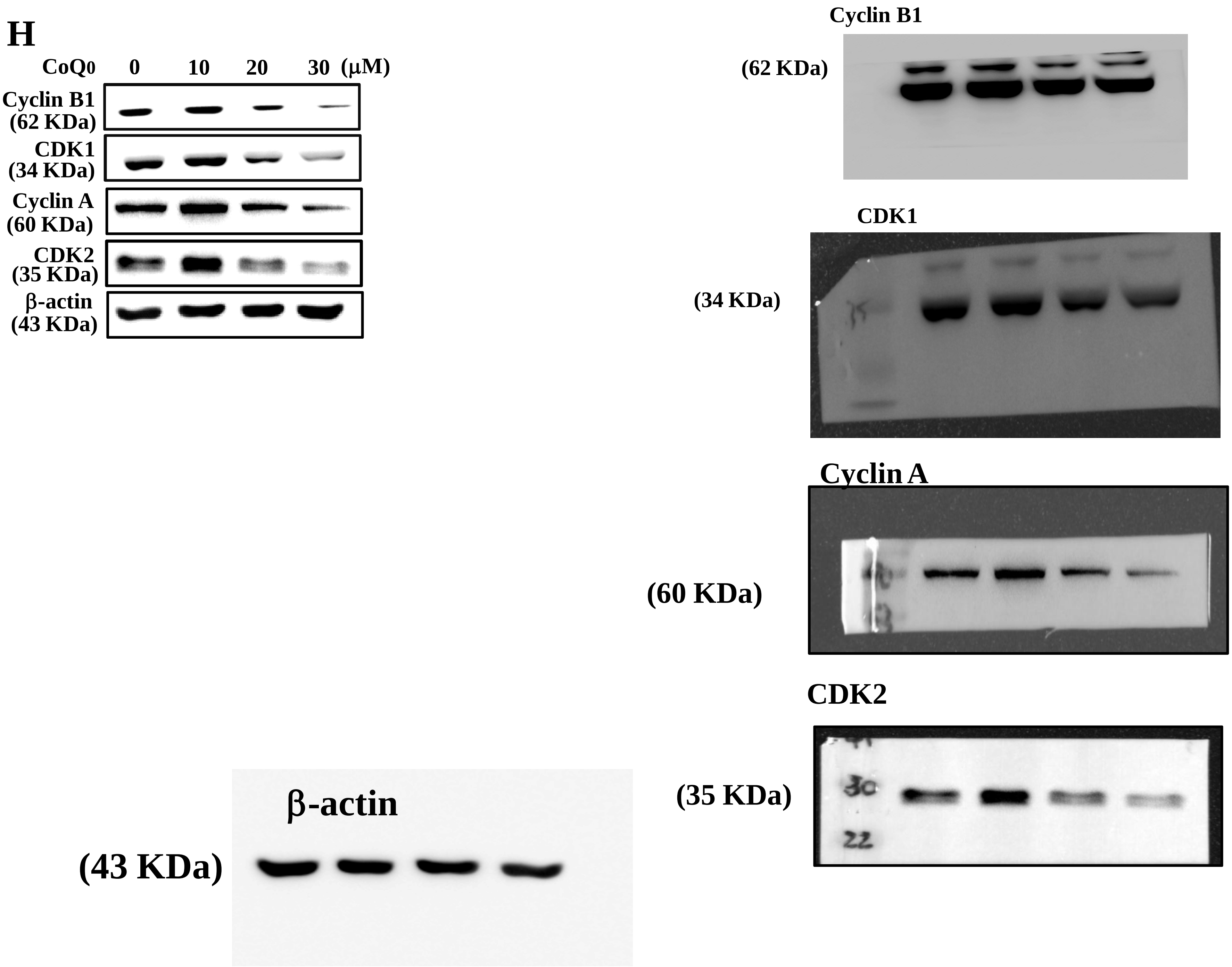
H
CoQ0 0 10 20 30 (μM)
Cyclin B1 (62 KDa)
CDK1 (34 KDa)
Cyclin A (60 KDa)
CDK2 (35 KDa)
β-actin (43 KDa)
Cyclin B1
(62 KDa)
CDK1
(34 KDa)
Cyclin A
(60 KDa)
CDK2
(35 KDa)
β-actin
(43 KDa)

Fig 1

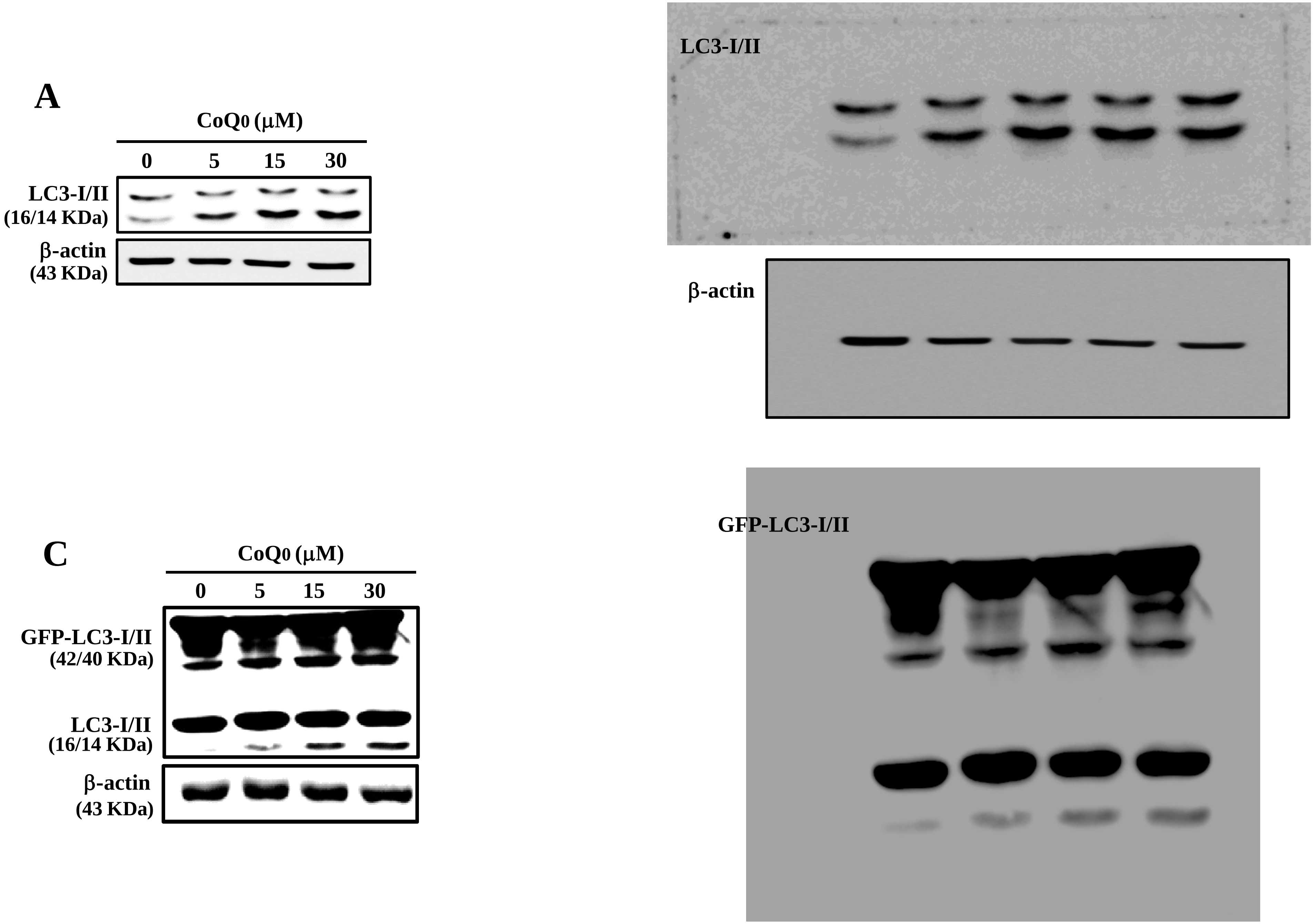
A
CoQ0 (μM)
0
5
15
30
LC3-I/II
(16/14 KDa)
β-actin
(43 KDa)
LC3-I/II
β-actin
C
CoQ0 (μM)
0
5
15
30
GFP-LC3-I/II
(42/40 KDa)
LC3-I/II
(16/14 KDa)
β-actin
(43 KDa)
GFP-LC3-I/II

Fig 3

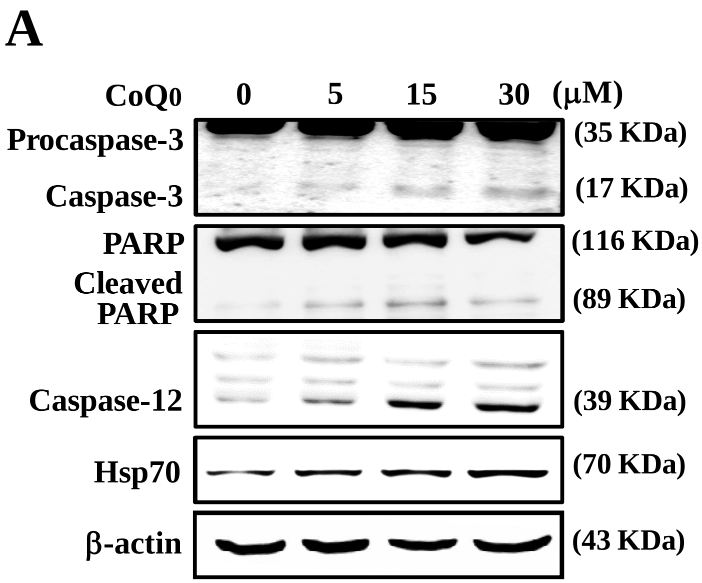
A
CoQ0 0 5 15 30 (μM)
Procaspase-3
Caspase-3
PARP
Cleaved PARP
Caspase-12
Hsp70
β-actin
(35 KDa)
(17 KDa)
(116 KDa)
(89 KDa)
(39 KDa)
(70 KDa)
(43 KDa)

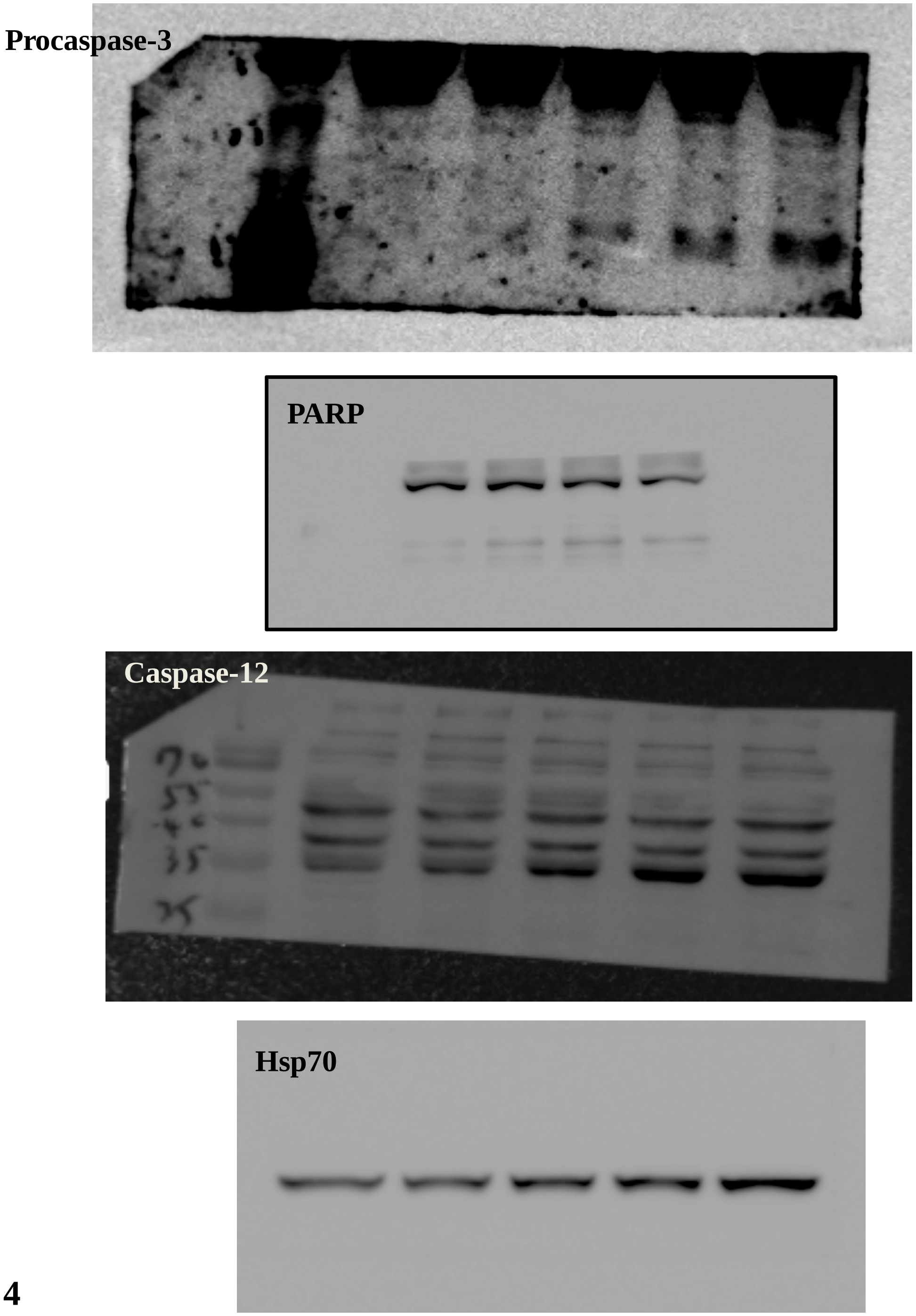
Procaspase-3
PARP
Caspase-12
Hsp70

Fig 4

C

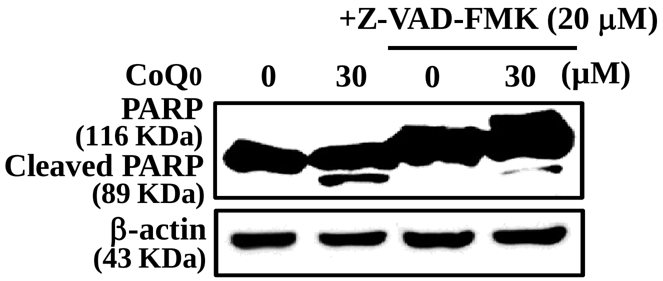
+Z-VAD-FMK (20 μM)
CoQ0 0 30 0 30 (μM)
PARP
(116 KDa)
Cleaved PARP
(89 KDa)
β-actin
(43 KDa)

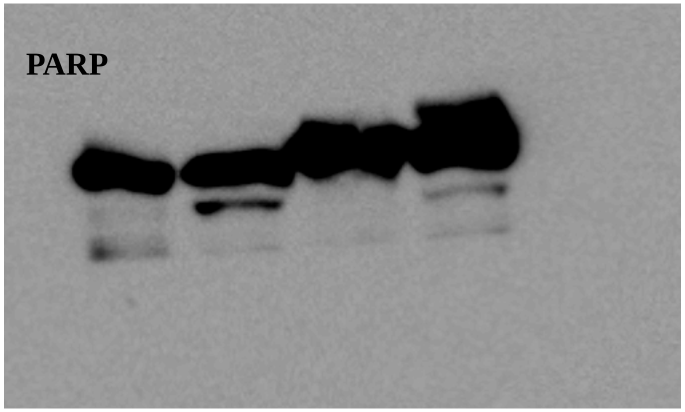
PARP

Fig 5

A

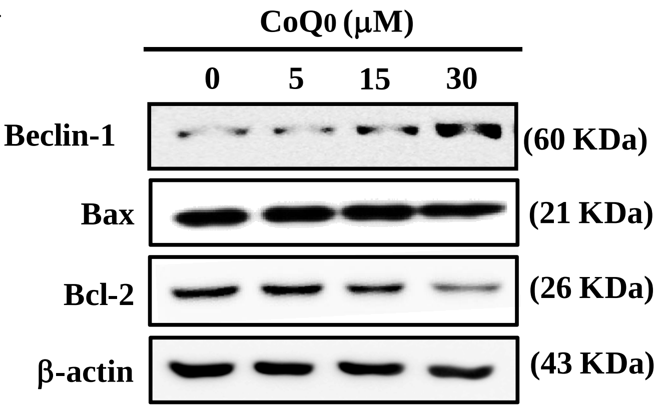
CoQ0 (μM)
0
5
15
30
Beclin-1
(60 KDa)
Bax
(21 KDa)
Bcl-2
(26 KDa)
β-actin
(43 KDa)

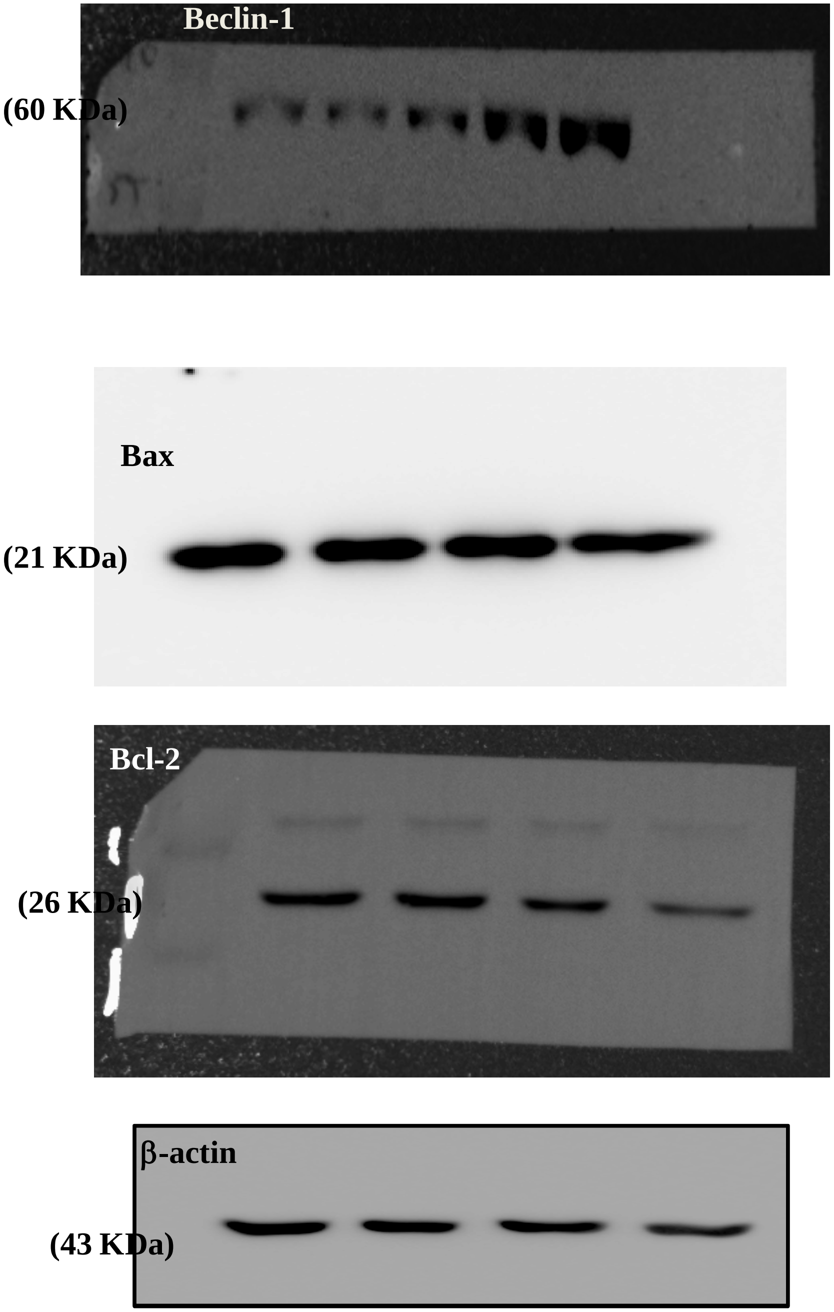
Beclin-1
(60 KDa)
Bax
(21 KDa)
Bcl-2
(26 KDa)
β-actin
(43 KDa)

Fig 6

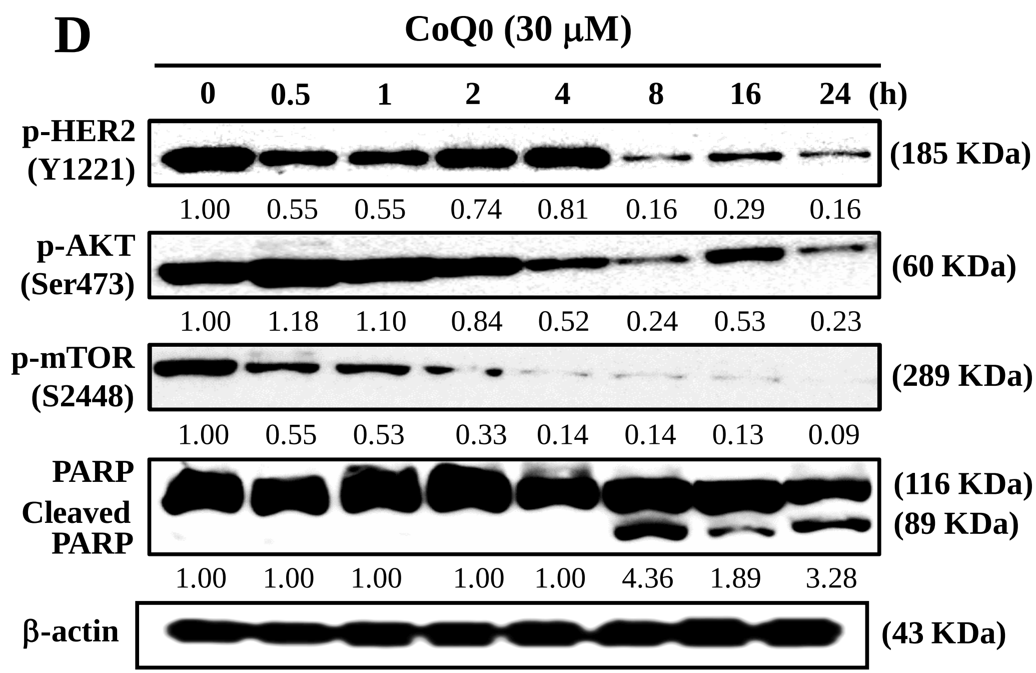
D
CoQ0 (30 μM)
0 0.5 1 2 4 8 16 24 (h)
p-HER2 (Y1221)
(185 KDa)
1.00 0.55 0.55 0.74 0.81 0.16 0.29 0.16
p-AKT (Ser473)
(60 KDa)
1.00 1.18 1.10 0.84 0.52 0.24 0.53 0.23
p-mTOR (S2448)
(289 KDa)
1.00 0.55 0.53 0.33 0.14 0.14 0.13 0.09
PARP
Cleaved PARP
(116 KDa)
(89 KDa)
1.00 1.00 1.00 1.00 1.00 4.36 1.89 3.28
β-actin
(43 KDa)

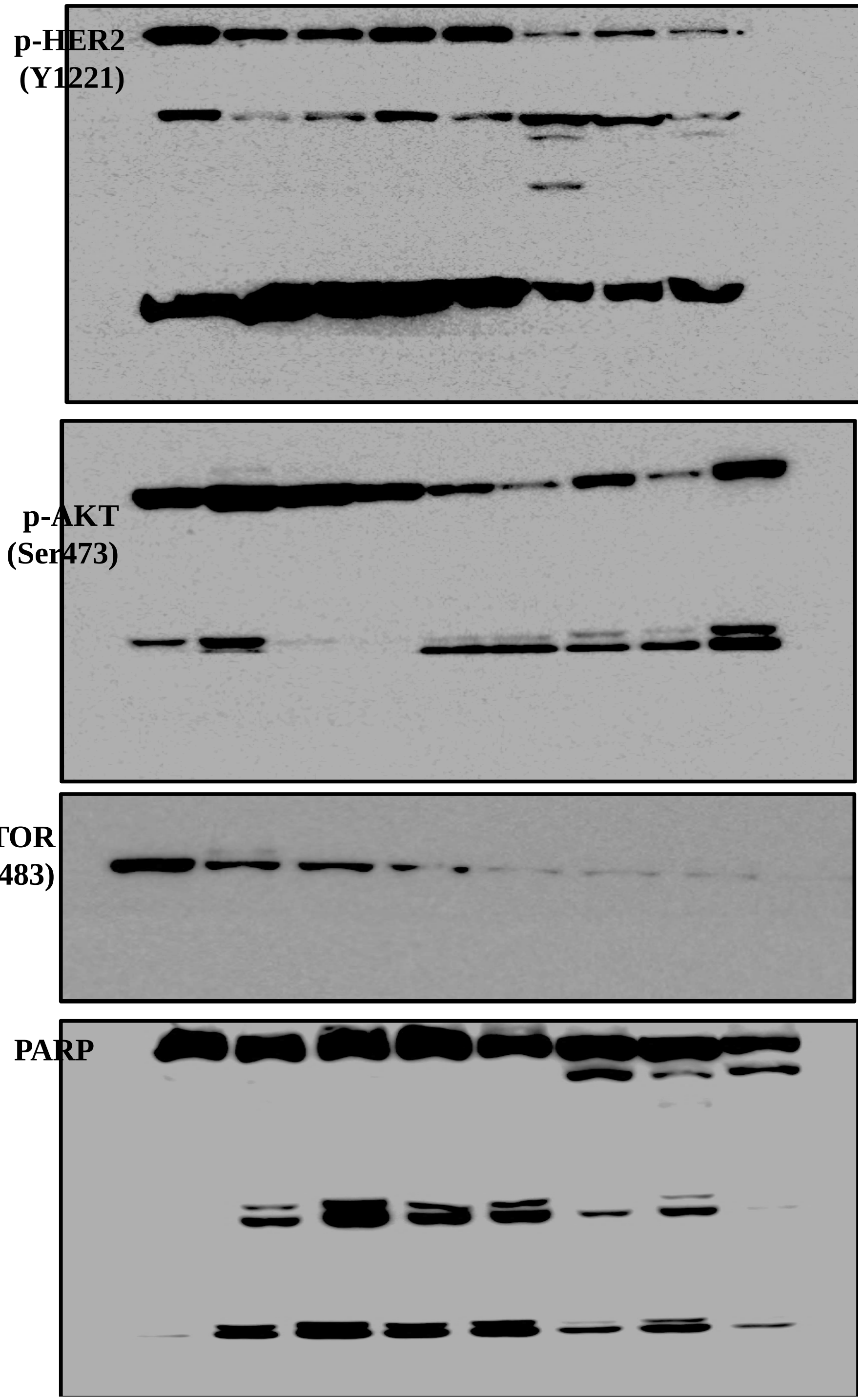
p-HER2 (Y1221)
p-AKT (Ser473)
p-mTOR (S24483)
PARP

Fig 6

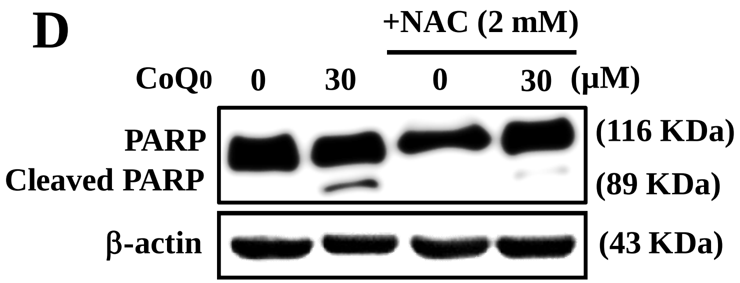
D
+NAC (2 mM)
CoQ0 0 30 0 30 (μM)
PARP
Cleaved PARP
β-actin
(116 KDa)
(89 KDa)
(43 KDa)

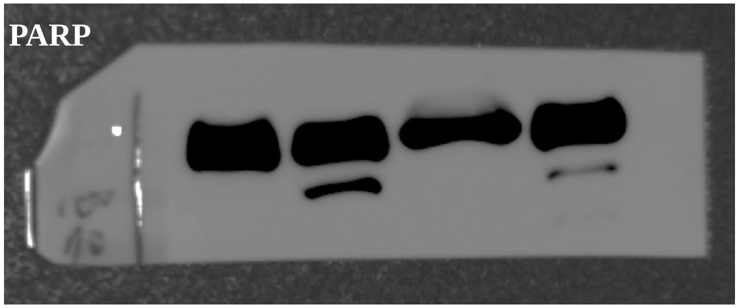
PARP

Fig 7

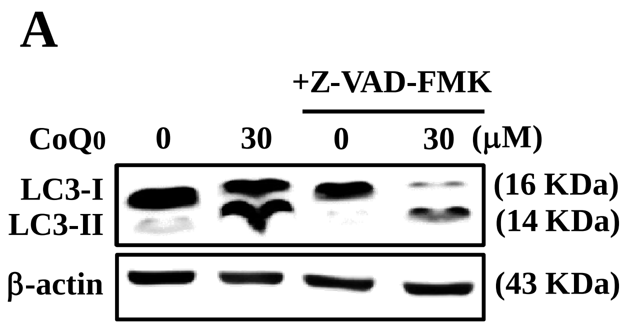
A
+Z-VAD-FMK
CoQ0 0 30 0 30 (μM)
LC3-I
LC3-II
(16 KDa)
(14 KDa)
β-actin
(43 KDa)

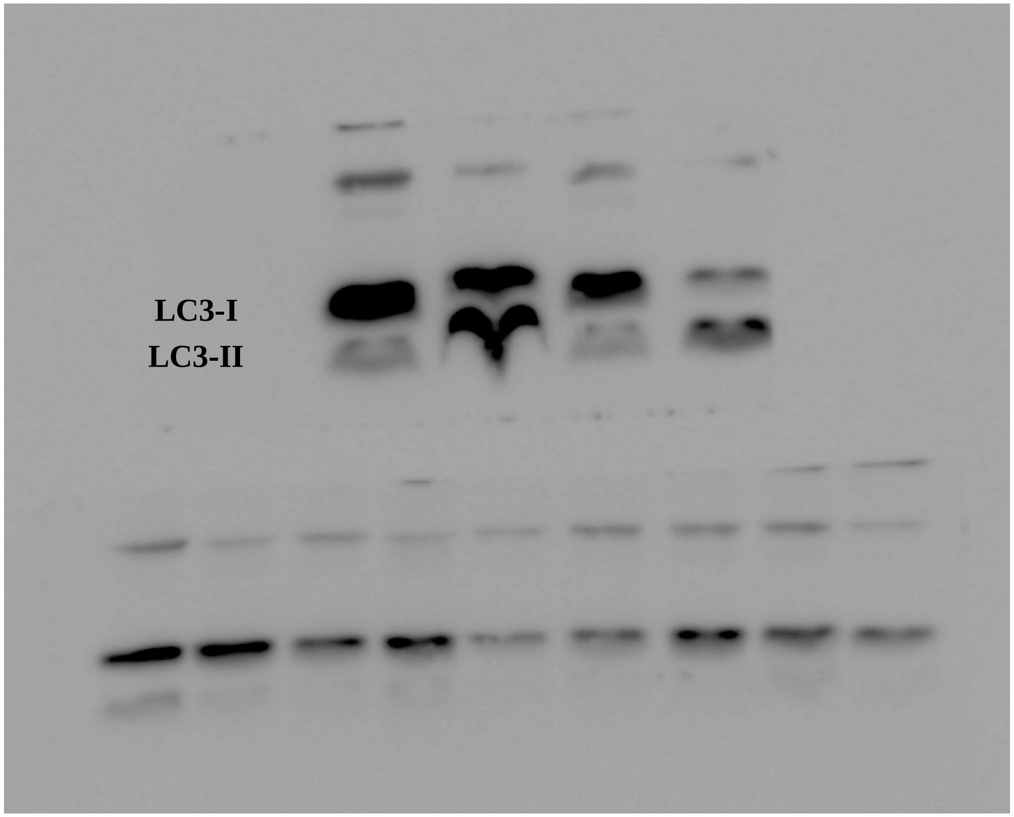
LC3-I
LC3-II

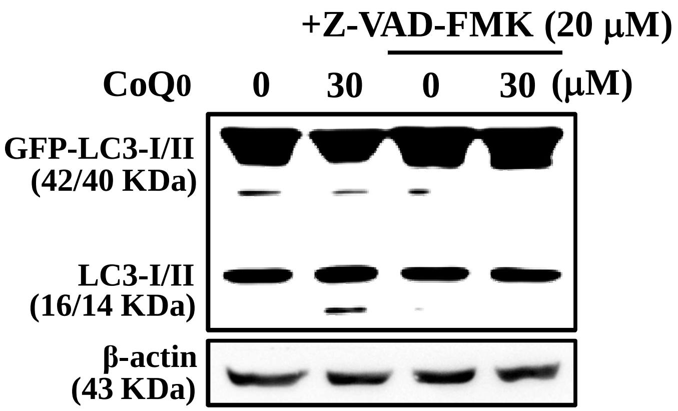
+Z-VAD-FMK (20 μM)
CoQ0 0 30 0 30 (μM)
GFP-LC3-I/II
(42/40 KDa)
LC3-I/II
(16/14 KDa)
β-actin
(43 KDa)

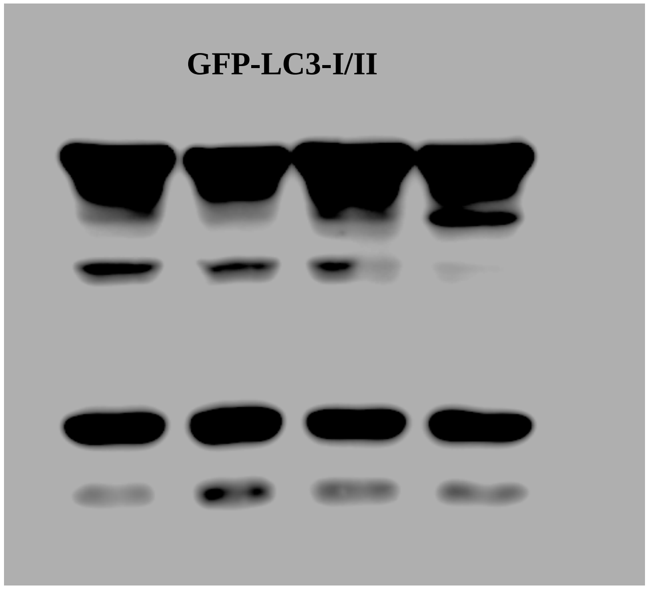
GFP-LC3-I/II

Fig 8

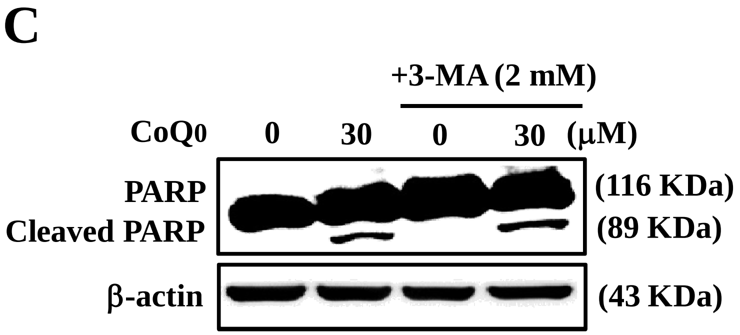
C
+3-MA (2 mM)
CoQ0 0 30 0 30 (μM)
PARP
Cleaved PARP
β-actin
(116 KDa)
(89 KDa)
(43 KDa)

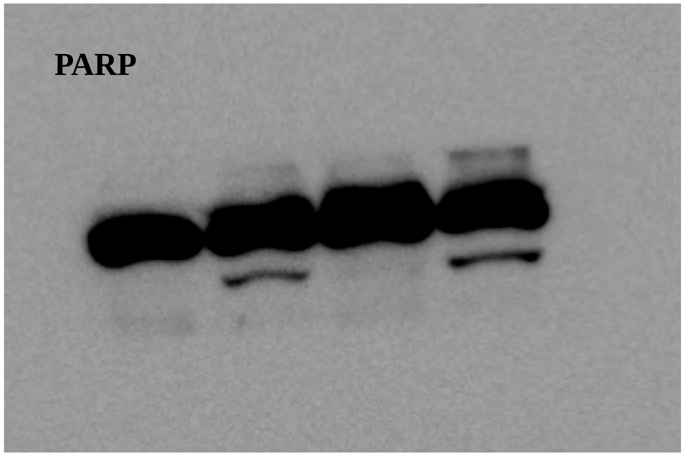
PARP

Fig 9
